# A neural architecture search optimized lightweight attention ensemble model for nutrient deficiency and severity assessment in diverse crop leaves

**DOI:** 10.1038/s41598-025-20124-4

**Published:** 2025-10-27

**Authors:** Sudhakar Muthusamy, Swarna Priya Ramu

**Affiliations:** School of Computer Science Engineering and Information Systems, Vellore Institute of Technology, Vellore, India

**Keywords:** Banana crop, Image classification, Micro-nutrient deficiency, Ensemble learning, Attention mechanism, Neural architecture search (NAS), Real-time application deployment., Abiotic, Computer science

## Abstract

The growth and productivity of banana crops are critically affected by micronutrient deficiencies, which are often difficult to detect at early stages. Lightweight deep learning models, optimized through neural architecture search (NAS) and attention mechanisms, are hypothesized to provide accurate and efficient classification of such deficiencies for real-time agricultural applications. In this study, multiple convolutional neural networks (CNNs) and mobile-friendly architectures, including ResNet50, VGG16, NASNetMobile, and MobileNet variants (V1, V2, V3), were evaluated using transfer learning on a curated banana leaf deficiency dataset. To improve robustness and prediction accuracy, modified classification layers and ensemble strategies–initially average ensembling and later a NAS-guided dynamic attention weighting mechanism were employed. This optimization resulted in a novel lightweight model, NASMobV2 (NASNetMobile + MobileNetV2), capable of both classifying nutrient deficiencies and assessing their severity levels. The proposed model achieved a validation accuracy of 98.57%, outperforming baseline and state-of-the-art counterparts in precision, recall, and F1 score. To improve generalization, banana crop diseases along with an additional Coffee crop dataset were included for evaluation. Finally, the practical utility of the model was demonstrated by deploying the trained system in both mobile and web applications, enabling farmers and agronomists to perform fast and accurate diagnostics directly in the field.

## Introduction

**Fig. 1:**
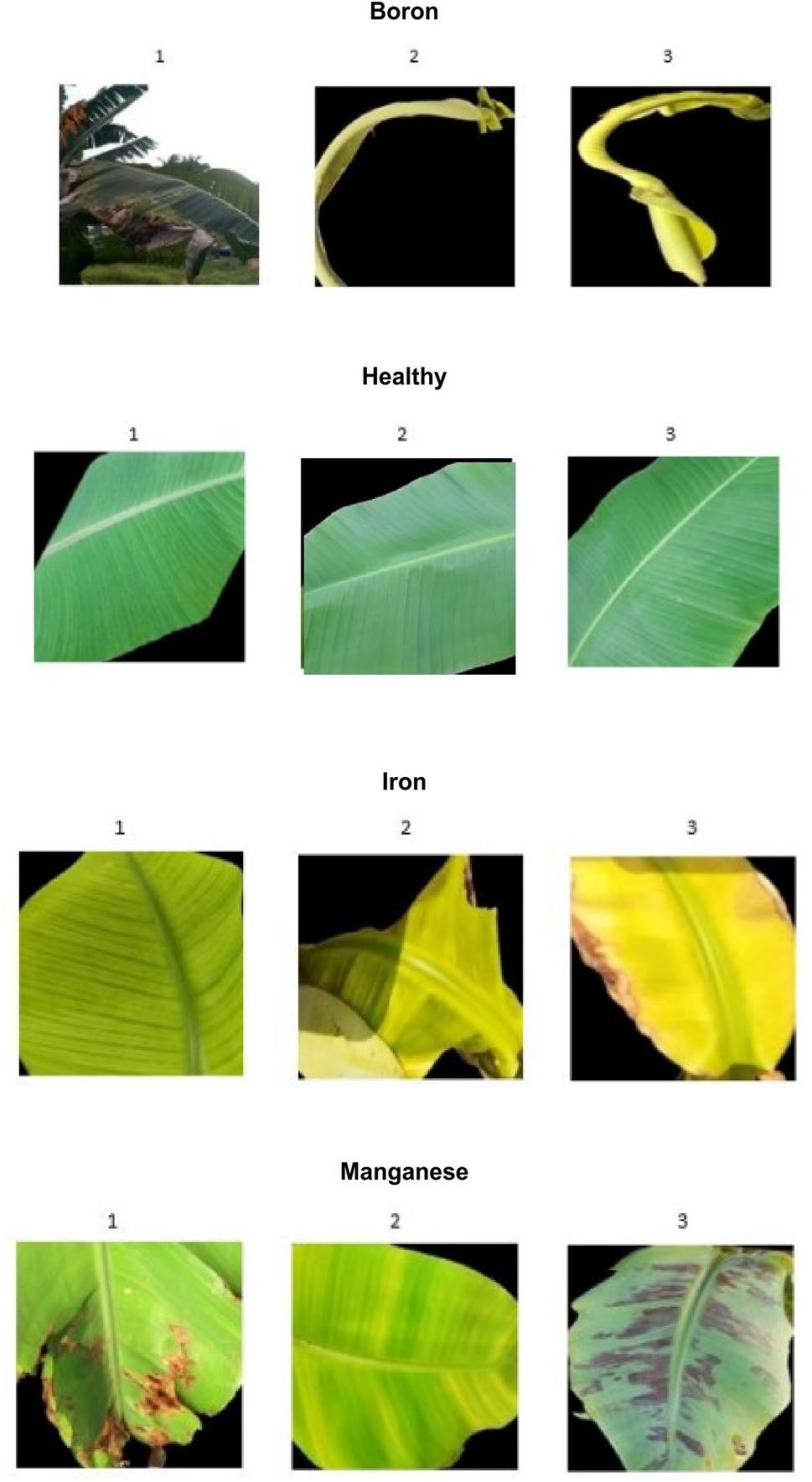
sample images of Banana Micro-Nutrient deficiencies from Mendeley dataset.

**Fig. 2:**
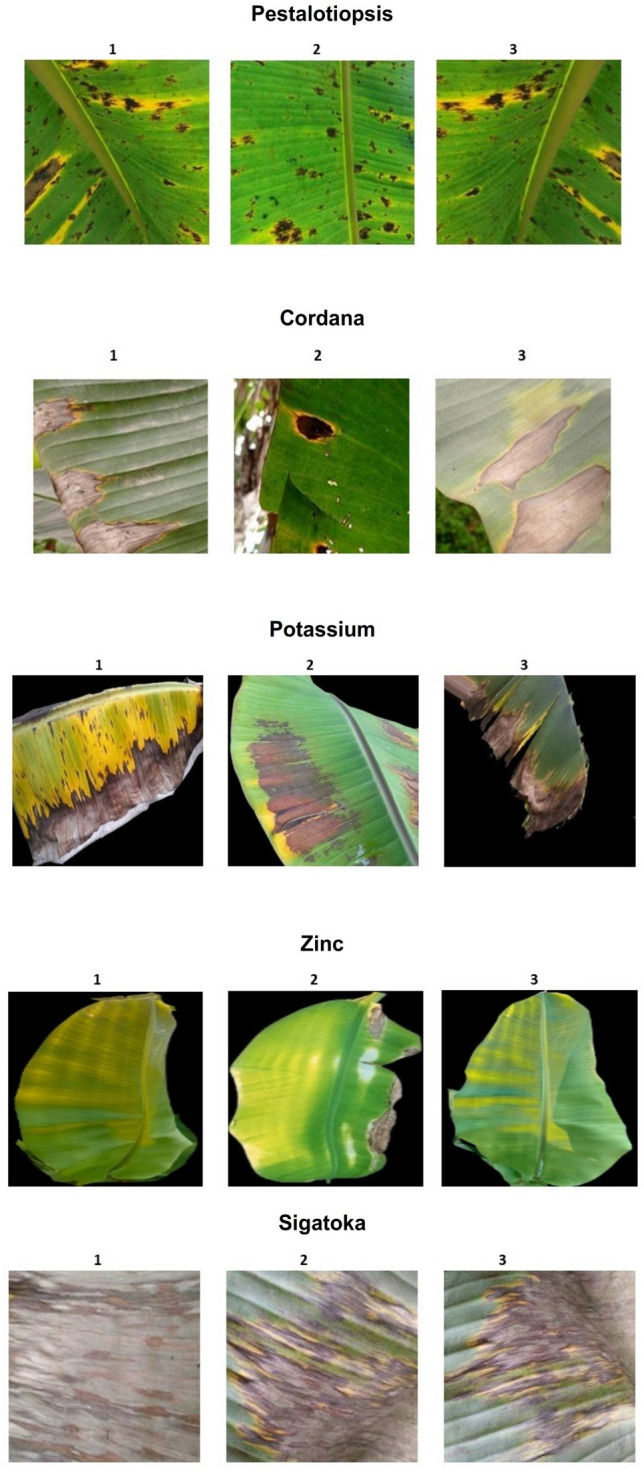
Additional sample images of Banana Micro-Nutrient deficiencies and diseases.

**Fig. 3:**
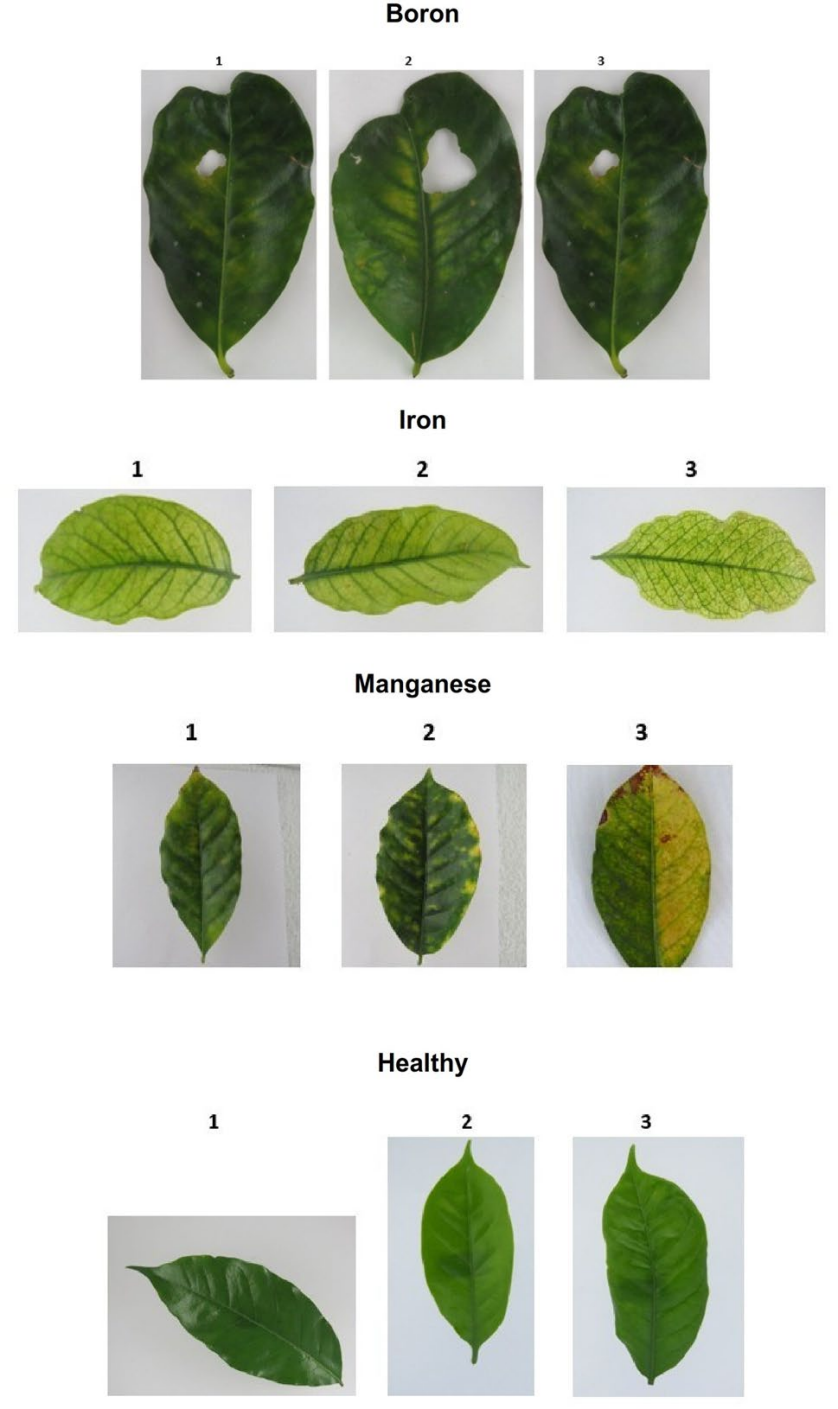
sample images of Coffee crop image deficiencies from Co-Leaf DB dataset.

Agriculture plays a vital role in the protection of human life, the survival of animals, and the maintenance of a balanced environment. It directly depends on key environmental factors such as water, sunlight, and soil nutrients. Among these, nutrient availability is critical, as both excess and deficiency negatively affect crop growth. Insufficient nutrients can reduce yield, weaken resistance to disease, and increase vulnerability to environmental stress. And the symptoms of deficiency appear different in different crops because the nature and biological qualities of the crops differ. Thus, the balance of crop nutrients ensures better productivity and supports human and animal health through improved food quality^1^. Crops are exposed to both biotic and abiotic stresses that cause production loss^2^. The expression of nutrient deficiency symptoms are linked to the mobility of mineral elements. Macronutrients like nitrogen (N), phosphorus (P), and potassium (K) are mobile, so symptoms appear on older leaves. In contrast, micronutrients such as boron (B), iron (Fe), manganese (Mn), zinc (Zn), and molybdenum (Mo) are largely immobile, so deficiencies are first visible on younger leaves. Other nutrients like sulfur (S) and calcium (Ca) are vital structural elements of plant cells and are not easily translocated. Common visible symptoms include chlorosis, necrosis, stunted growth, premature leaf and bud drop, and inhibited cell division. Chlorosis, for instance, occurs due to a lack of minerals like N, K, Mg, S, B, Fe, Mn, Zn, or Mo^3^, while necrosis can result from Ca, Mg, Cu, or K deficiencies. In many cases, it is difficult to distinguish deficiencies such as nitrogen, magnesium, or sulfur due to overlapping symptoms. Micronutrient chlorosis, caused by deficiencies of boron, iron, manganese, or zinc, further complicates diagnosis. This complexity often confuses farmers, who struggle to differentiate between macro and micro-nutrient deficiencies. The Figs. 1, 2 and 3 represent the sample images of deficiencies with diseases as well as healthy leaves belonging to banana and coffee crops.

India is the world’s largest producer of bananas, contributing significantly to the global market. Tamil Nadu, Karnataka, and Maharashtra are leading states in terms of banana cultivation and production^4^. Given its importance, accurate nutrient monitoring in banana crops is crucial. Traditionally, nutrient analysis relies on soil testing, leaf and stem testing, remote sensing, or water testing. However, these conventional methods are destructive, time-consuming, and require laboratory facilities. Farmers therefore need quick, reliable, and non-destructive alternatives. Recent advancements in computer vision, machine learning (ML), and deep learning (DL) provide effective solutions for nutrient deficiency detection. Using sensors, Unmanned aerial vehicle (UAV)’s , Internet of things (IoT) systems, and imaging devices, crop monitoring can be performed with higher precision. However, techniques like spectral imaging or optical sensors are expensive. In contrast, computer vision models can identify nutrient deficiencies using leaf image features such as color, texture, venation, and shape. With the support of convolutional neural networks (CNNs), transfer learning, and attention mechanisms, these models achieve reliable performance.

**Fig. 4:**
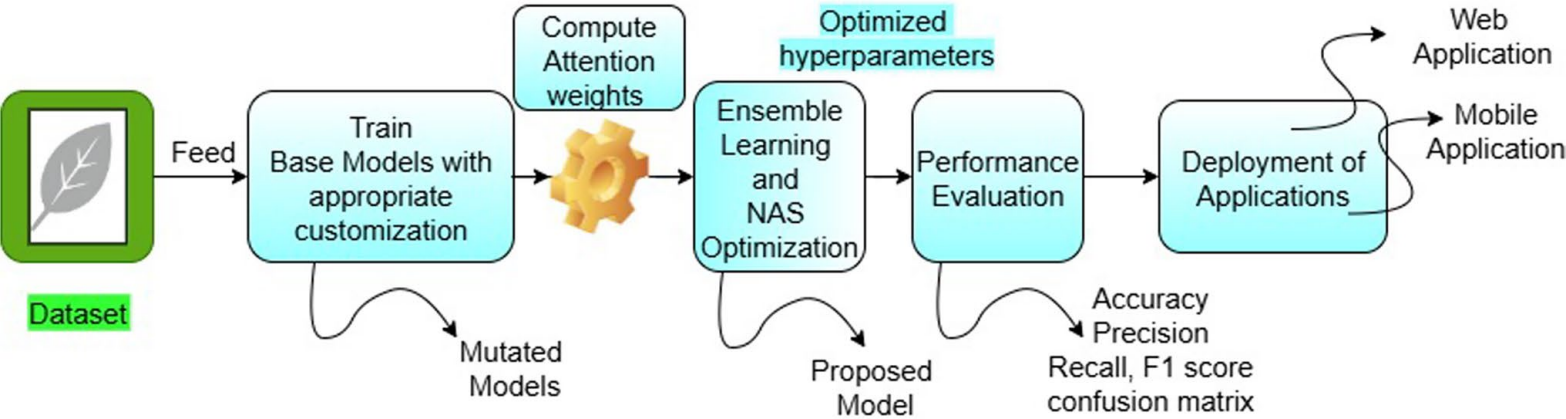
Overview of the proposed model.

In this study, the focus is to detect banana crop nutrient deficiencies through leaf image analysis. The proposed method employs an attention-based NAS-optimized ensemble learning approach, where mutation of CNN layers and tuned hyper-parameters enhance classification accuracy. The model not only identifies specific nutrient deficiencies but also estimates severity levels, making it more effective for real-time decision support. To ensure practical usability, the methodology was implemented as both a web application (using Streamlit and GitHub) and a mobile application, enabling farmers to test crop leaves under real-world conditions such as varying canopy structures, illumination, and rotation. This work aims to provide a robust and scalable solution that combines methodology implementation, CNN-based feature learning, transfer learning, and ensemble techniques, ultimately supporting farmers with accurate nutrient diagnosis and actionable recommendations. The overview of the proposed model is depicted in Fig. 4.

### Significance of this work

- To the best of our knowledge, this is a significant work contributing as a novel ensemble of two transfer learning(TL) models being performed as a mobile based diagnosis using light weight deep learning models for micro nutrient deficiency diagnosis of banana crops.
- The performance metrics were evaluated for the modified pre-trained deep learning models and further extended to create an ensemble model that provides better feature extraction.
- Our proposed model finds optimal architecture combinations that improve the model classification efficiency with less complexity in terms of parameters and training duration.
- We estimated the severity levels of the deficiencies from the input images to further improve the classification model.
- A web application implemented using a streamlit app, github and a mobile application was also developed for testing the real-time image classification of crop nutrient deficiency.
- The improvement of this application serves as a more robust model for different real-time conditions, such as the canopy structure of crops, illumination and rotation.

### Organization of the paper

The remainder of this paper is organized as follows. The Literature study describes the related work done to identify crop nutrient deficiencies. The next section explains the details of the dataset resources used and the proposed methodology implementation, along with the details of the deployment of deep learning(DL) models as a web and a mobile application. The following section gives the experimental results and discussion. The final section presents the conclusions and future work.

## Literature study

This content expresses the related work done by different researchers worldwide with regard to nutrient deficiency identification. They computed the nutrient content of a crop through the establishment of machine learning(ML) / DL models. Few studies have combined traditional machine learning methods with feature selection and reduction strategies to reduce computational complexity. Paymode et al.^5^ proposed a mobile application, Agrodeep to capture leaf images in crops with six types of affected diseases. The proposed convolutional neural network(CNN) model outperformed and achieved the comparatively highest accuracy of 97%. The technique used was a faster region- based CNN (faster R-CNN). Chen et al.^6^ created a stack of different versions of light weight MobileNet based CNNs which include Mobile-DANet, SE-MobileNet and MobileNetV2 to form a significant network called Es-MbNet to differentiate between plant diseases. They attained useful results in their work, but the differences and diversity of the images used were limited. A two-level stacked ensemble neural network model, called Es-MbNet, was suggested after a plant disease dataset was gathered in the first stage. The model was able to acquire the basic features of crop disease photos first and then progressively shift its focus to finer details. Gunawardena et al.^7^ proposed a mobile device based edge- computing architecture model for human eye tracking. The dataset was collected using Gazecapture software. A web application, mobile device, edge servers, and cloud data center comprise the proposed architecture. Four lightweight CNNs- Lenet, AlexNet, MobileNet V3, and ShuffleNet V2, were used for comparative performance analysis. Memory, energy usage, inference, and communication time were among the parameters measured. According to their analysis, the best models with the least amount of error are shuffleNet V2 and MobileNet V3. Shrimali^8^ developed a widely accessible mobile application called PlantifyAI based on a light weight CNN model. They were able to classify 26 crop diseases in 14 different types of species. They compared 16 CNN models, among which the MobileNet V2 along with the Canny edge detection filter, which resulted the highest classification accuracy of 95.7 % and F1 score of 96.1%. Haris et al.^9^ suggested a method to identify nutrient deficiencies in guava leaf images. The dataset was created using a mobile application based on deep learning. Approximately 664 photos showing deficiencies in nitrogen, phosphorus, magnesium, and manganese were gathered from various guava farms throughout the state of Karnataka. The data were pre-processed after collection to correlate and adapt the feed to the proper deep learning model. The suggested approach yielded an accuracy of 87% overall, with individual class accuracy of 86.9% for Mn, 91.2% for Mg, 79.2% for P, and 91.5% for healthy.

Munir et al.^10^ proposed a new graph based sparse attention mechanism based architecture for vision applications on mobile devices. The hybrid mobile ViG model that integrates the CNN-GNN architecture achieved better results than the existing ViG models including MobileNetv2. Hussain et al.^11^ investigated state-of-the-art CNN-based machine learning applications for plant identification in mobile devices. The authors experimented with MobileNet transfer learning with two versions, MobileNet V2 and v3, to improve the recognition accuracy. Fine-tuning was performed by freezing the weights of the base layers and modifying the top layers making them suitable for the task. The performance parameters were evaluated and found that MobileNet V3 had the highest accuracies of 89% and 91% at 10 and 100 epochs, respectively. In addition, they believed that the accuracy could be increased within 20 epochs using data augmentation techniques. Nagi and Tripathy^12^ designed a light weight six layer CNN model architecture for grapevine disease classification. There were three disease classes and one healthy category. With respect to classification accuracy, the proposed model outperformed Alexnet, MobileNet, and VGG16, achieving 98.4 % on the test dataset.

El-bana et al.^13^ evaluated the potential of wavelet pooling in light weight CNN models for improving the data efficiency. Authors investigated with many datasets and based on the performance metrics, and concluded that by using wavelet based pooling methods exceeded the data saving efficiency and classification accuracy by 30% compared to the traditional pooling techniques. With regard to a medical application, Chen et al.^14^ studied the best performing light weight CNN models, such as DenseNet, MobileNetV2 and NasNetMobile to create an ensemble model usage in a smartphone application that recognizes middle ear diseases or categorizes eardrum disorders.The Measurement parameters such as the class activation map, were used to identify key classification features. The proposed model based on edge computing achieved a recognition accuracies of 97.4% and distinguishing accuracy of 98.9 %. Xie and Liao^15^ proposed a compact classification model by integrating the inductive bias ability in a CNN with the global modeling feature representation capability of ViT (vision transformer). They used three datasets, namely CIFAR10, CIFAR100 and Stanford cars, to evaluate the intended model. The proposed model reached top 1 accuracy of 89.87 % which is improved of 3.32 %. With fewer parameters, an Efficient ViT can extract features as a result of its combination of CNN and ViT properties. Wu et al.^16^ implemented a portable CNN based system called Deep BarkID to identify tree species from its bark image through knowledge distillation. The authors applied the vanilla response based mimic teacher student knowledge model for their obtained dataset to achieve the best performance The proposed model attained 96.12 % classification accuracy between ten tree species in USA. Liu et al.^17^ developed a modified version of a light weight CNN model for detecting the severity of crop diseases. The authors performed experiments with three other models: ResNet50, Xception and MobilenetV2. They implemented a multi-scale convolution kernel to accurately distinguish the features. The dataset used for evaluation was obtained in a laboratory environment. The overall performance metrics were compared with those of the proposed model which showed recognition accuracy of 91.94 % which was 3.02% higher than squeezenext model. However the major limitation of this study was that the performance was not tested under real-time conditions.

Deb et al.^18^ developed a novel CNN architecture called ConvPlant-Net, which is used to effectively detect crop diseases by combining a learning layer with both high-level and low-level features with a depthwise separable convolutional, two-dimensional transpose layer. Three crops - potatoes, peppers, and tomatoes, were used to test the model. The images were obtained from a publicly accessible plantvillage dataset. The efficiency of the model was considered better because it carried only 31,998 trainable parameters. The highest accuracy was attained on the potato crops at 97 %, following which the tomato crop classification had an accuracy of 96%. Prasad et al.^19^ implemented a modified compressed version of the baseline CNN architecture of MobileNetV3 for mobile based resource -constrained platform applications. The authors made changes in the convolution layers, activation function, and number of expansion filters and the model size was reduced 84.96 % with improved validation accuracy of 0.2 %. Another group of authors^20^ developed an AI based mobile application to detect banana plant diseases and pests. Shruthi et al.^21^ proposed a feature extraction technique ResNet2 using light weight CNN models for the classification of 14 apple varieties. The dataset was obtained from GitHub and Kaggle online repositories and comparative performance evaluation was performed among the already trained benchmark models on the ImageNet database, such as ResNet50, VGG16, MobileNet and EfficientNetB0. In this study, a stem block and two residual blocks were used for the feature extraction. The obtained accuracy results were 99.8% during training phase, 98.72 % validation and testing accuracy of 99.59 % which outperformed the considered models. An ensemble transfer learning approach was suggested for nutrient deficiency identification and yield loss prediction in this study^22^. Two experiments were conducted to assess the proposed approach using rice and groundnut datasets. The proposed ensemble transfer learning methodology achieves accuracy rates of 94% and 99%, respectively. The authors^23^ proposed a hybrid meta-heuristic approach–named PSOBER–that optimizes Convolutional Neural Network (CNN) and Deep Belief Network (DBN) parameters using a combination of Particle Swarm Optimization (PSO) and the Al-Biruni Earth Radius (BER) algorithm. The method was applied to detect oral cancer in medical images, achieving a classification accuracy of 97.35%, which surpassed several competing models. A comprehensive article^24^examined the integration of drones and Internet of Things (IoT) technologies in sustainable agriculture, emphasizing their potential to enhance efficiency and support smallholder farmers. The study identifies key challenges, including connectivity issues in rural areas, high initial costs, and the complexity of integrating various IoT systems. To address these, the authors propose solutions such as leveraging satellite communication, LoRa networks, and mesh networking to improve data transmission. The research underscores the importance of affordable technology and data-driven strategies in overcoming barriers and promoting sustainable farming practices. The authors^25^ proposed an optimized voting classifier for classifying weed and wheat images captured by sprayer drones. The classifier integrates neural networks (NNs), support vector machines (SVMs), and K-nearest neighbors (KNN), optimized using a hybrid of sine cosine and grey wolf optimizers. Features are extracted via transfer learning from AlexNet, with significant features selected using a novel algorithm. The approach achieved a detection accuracy of 97.70%, F-score of 98.60%, specificity of 95.20%, and sensitivity of 98.40%. The authors^26^ propose a hybrid training algorithm for neural networks that combines Invasive Weed Optimization (IWO) with Differential Evolution (DE) to enhance learning efficiency and accuracy. The approach was applied to benchmark datasets and demonstrated improved performance compared to standard training methods, achieving higher classification accuracy and faster convergence. The authors^27^ propose a dynamic programming-based pruning algorithm to optimize classifier ensembles. Evaluated on benchmark datasets, the pruned ensembles achieved up to 95–97% classification accuracy while significantly reducing the number of classifiers, demonstrating improved computational efficiency without loss of performance. In a work^28^, the authors present a method for designing optimal classifier ensembles using cooperative game theory to assign weights based on classifier contributions. Applied to benchmark datasets, the approach improved ensemble accuracy by 3–5% compared to traditional unweighted ensembles, demonstrating enhanced classification performance and robustness.In a significant study,^29^ the authors proposed a modified metaheuristic algorithm to optimize convolutional networks for apple tree leaf disease detection. The method achieved higher classification accuracy and faster convergence compared to standard CNN training, demonstrating the benefits of optimization for plant leaf classification task. A novel study^30^ introduced a multiswarm firefly algorithm to optimize hyperparameters for plant classification tasks. The method demonstrated improved classification accuracy over traditional approaches, highlighting the effectiveness of swarm intelligence in enhancing plant image classification. The summary of related works on nutrient deficiency and disease identification using ML,DL and AI-based approaches is listed in Table 1.

**Table 1:** Summary of related works on nutrient deficiency and plant disease identification using ML, DL, and AI-based methods.

References	Methodology	Crop & Dataset	Deficiency / Disease	Results	Advantages	Disadvantages
Nagi and Tripathy^12^	Lightweight 6-layer CNN	Grapevine dataset	3 diseases + healthy	98.4%	Simple, efficient	Limited classes
El-Bana et al.^13^	Wavelet pooling with CNNs	Multiple datasets	General classification	+30% efficiency vs. pooling	Data-efficient	Controlled datasets
Wu et al.^16^	Deep BarkID (distilled CNN)	Bark images (10 species)	Tree species ID	96.12%	Portable, efficient	Bark-only scope
*Deep Learning (DL)-based Approaches*						
Paymode et al.^5^	Faster R-CNN (Agrodeep mobile app)	Tomato, 6 diseases	Disease detection	97%	High accuracy, real-time mobile deployment	High computation, crop-specific
Chen et al.^6^	Es-MbNet (stacked MobileNet variants)	Plant disease dataset	Plant diseases	Strong feature extraction	Good hierarchical learning	Dataset diversity limited
Shrimali^8^	MobileNetV2 + Canny edge filter (PlantifyAI app)	14 species, 26 diseases (PlantVillage)	Crop diseases	95.7%, F1 = 96.1%	Large coverage, robust deployment	Dataset synthetic, not real field conditions
Haris et al.^9^	DL model on guava dataset	Guava leaf images (664 samples)	N, P, Mg, Mn deficiency	87% overall (classwise: Mn 86.9, Mg 91.2, P 79.2, Healthy 91.5)	First work on guava nutrient deficiency; mobile data collection	Low dataset size; moderate accuracy
Munir et al.^10^	CNN–GNN hybrid (MobileViG)	Vision datasets	General plant vision tasks	Better than MobileNetV2 and ViG baselines	Stronger feature modeling, sparse attention	Complex hybrid; high training demand
Hussain et al.^11^	MobileNet V2/V3 (transfer learning)	Plant identification datasets	General plant ID	V3: 91%	Lightweight, mobile-efficient	Needs augmentation for better generalization
Chen et al.^14^	Ensemble (DenseNet + MobileNetV2 + NasNetMobile) smartphone app	Medical (ear diseases)	Eardrum classification	97.4% recognition, 98.9% distinguishing accuracy	Ensemble + edge computing = robust	Focused on medical, not agriculture
Xie and Liao^15^	EfficientViT (CNN + Transformer)	CIFAR10, CIFAR100, Stanford Cars	General image classification	89.87% (Top-1)	Combines CNN with ViT strengths	Tested on generic datasets, not agriculture
Liu et al.^17^	Modified lightweight CNN	Lab dataset (crops)	Disease severity	91.94%	Improved feature extraction with multi-scale kernels	Not tested in real field conditions
Deb et al.^18^	ConvPlant-Net (lightweight CNN)	PlantVillage (potato, pepper, tomato)	Crop diseases	97% (potato), 96% (tomato)	Few trainable params (31,998)	Limited to 3 crops, PlantVillage bias
Kavyashree et al.^19^	Compressed MobileNetV3	Resource-constrained mobile apps	General classification	Model size $\downarrow$ 84.96%, accuracy $\uparrow$ 0.2%	Optimized for mobile hardware	Minor accuracy improvement only
Shruthi et al.^21^	ResNet2 (feature extraction)	Apple varieties (GitHub + Kaggle datasets)	14 apple varieties	99.8% train, 98.72% validation, 99.59% test	Very high accuracy, ensemble feature learning	Dataset bias; limited crop coverage
*AI-based Approaches*						
Selvaraj et al.^20^	Tumaini mobile app (AI + CNN)	Banana crop images	Diseases + pests	90–99%	Practical field-ready AI tool	Limited to banana, disease focus not nutrient
Venkatesh et al.^22^	Ensemble transfer learning	Rice & groundnut datasets	Nutrient deficiency + yield loss	Rice: 94, Groundnut: 99	Strong ensemble; covers deficiencies + yield	Dataset-limited; only 2 crops

### Research gaps

- Existing research on plant stress detection has largely concentrated on disease identification using CNN and ViT-based models. However, nutrient deficiency detection – particularly micronutrient deficiencies such as Boron, Iron, and Manganese – has received limited attention.
- Moreover, integration of self-supervised or attention-based mechanisms that can improve fine-grained feature extraction with small datasets.
- Most available approaches either require extensive pixel-level annotations or fail to provide severity grading, making them impractical for real-world deployment.

Hence, a clear gap exists in developing lightweight, attention-enhanced models that can automatically segment leaf regions and classify nutrient deficiencies, even under limited annotated data. This study addresses this gap by contributing a novel ensemble of transfer learning(TL) models being performed as a mobile based diagnosis using light weight deep learning models for micro nutrient deficiency diagnosis.

## Resources and implementation tools

This paper proposes an ensemble model using NAS optimization for diagnosing the major micro nutrient deficiencies, such as boron, iron and manganese, in banana crops. The Data were collected from the publicly available fully labelled Mendeley dataset of banana crops which comprises eight micro nutrient deficient and healthy leaf images. The dataset was used to train each classifier model, and the models were then assessed for performance metrics such as accuracy, precision, recall, confusion matrix, and F1-score analysis. Based on the individual model performance results obtained, we selected the best models for integration to improve accuracy and reduce model complexity in terms of training time and space requirements. The computing hardware of the Dell system comprises an Intel i5 11th generation processor, 24GB RAM, and 2 GB NVIDIA MX 450 GPU. The Software tool used was the Jupyter notebook on the Windows operating system platform. Two applications- one for the web and one for mobile have been developed for testing real- time images for the diagnosis of crop nutrient deficiency. The web application was developed using **Streamlit (Release Version 1.29, 2023)** (https://streamlit.io) and deployed via both GitHub (https://github.com/msu1316/Deploy) and Streamlit Cloud (https://microbanana.streamlit.app/). The mobile application was implemented and tested using **Android Studio (Giraffe Version 2022.3.1, API Level 34)** (https://developer.android.com/studio). For demonstration purposes, the running mobile application was captured using the device’s inbuilt screen recorder.

### Study area, dataset details and pre-processing

The public dataset from Mendeley comprises banana leaf images exhibiting various nutrient deficiencies across cultivars such as Rasthali, Musa acuminata (Dwarf Cavendish), Robusta, Poovan, Elakkibale, and Monthan. These images were captured under diverse environmental conditions, including bright sunlight and low-light scenarios, from different regions of Karnataka State, India, and are available in both .jpg and .png formats^31^. From this source, selected 5434 images representing multiple micro-deficiencies (boron, iron, manganese, and zinc) along with macro-deficiency (potassium) including diseases and healthy leaves were chosen. To further strengthen the robustness of the model in real-world scenarios, additional banana leaf images were collected from agricultural fields within the VIT University (Vellore, India) campus, where crops were cultivated in a 30-cent area and exposed to natural conditions such as sunlight, rainfall, and pest activity. Pre-processing steps included scaling adjustments for deployment on mobile deep learning frameworks, along with manual verification to ensure accurate mapping of deficiency symptoms. Data augmentation techniques–such as rescaling, brightness variation, rotation, horizontal flipping, and zooming–were applied using Augmentor libraries and Keras ImageDataGenerator to mitigate overfitting. Post-augmentation, each class contained 1000 images, resulting in 6000 samples for deficiency detection and 3,000 for disease classification. In the case of coffee, the curated dataset of 2000 images were chosen, equally distributed across four classes (500 images per class), combining both publicly available samples and our own annotated data. The augmentation parameters used were: rescaling factor = 1./255, brightness range = [0.8, 0.8], and zoom range = [0.5, 0.5]. The dataset images were originally sized at 256 $\times$ 256 pixels, with a total storage size of 52.9 MB. The description of the dataset is listed in Tables 2 and 3. The data was divided into 80% for training and 20% for validation, while images were resized to 224 $\times$ 224 pixels for compatibility with lightweight CNN architectures. To evaluate model generalization, approximately 300 real-time images unseen by the model and 5% of the validation set were reserved for testing.

**Table 2:** Description of banana dataset from Mendeley.

Sl. No	Deficiency category	No. of original samples	No. of Augmented samples	Total No. of samples after augmentation
1	Boron	800	200	1000
2	Iron	860	140	1000
3	Healthy	950	50	1000
4	Manganese	840	160	1000
5	Zinc	713	287	1000
6	Potassium	381	619	1000
*Disease Category*				
7	Cordana	400	600	1000
8	Pestalotiopsis	173	827	1000
9	Sigatoka	473	527	1000

**Table 3:** Description of Coffee Dataset (Co-Leaf DB).

Sl. No	Deficiency category	No. of original samples	No. of Augmented samples	Total No. of samples after augmentation
1	Boron	273	227	500
2	Iron	298	202	500
3	Healthy	244	256	500
4	Manganese	271	229	500

### Transfer learning using pre-trained models

The principle of the transfer learning strategy is to apply learning from a task in one domain that can be used to solve problems in different domains. Here, the weights in a specific pre-trained model are retained, except for the classification stage which is modified to meet the specific problem domain. This means that the neural network architecture does not need to be built from scratch; instead some modifications or mutations could be performed to adapt to our particular domain. It helps to attain time efficiency by using less processing time of model weights because the model has already been trained on large natural image databases such as ImageNet. Here, two popular TL models namely NASNetMobile, MobileNetV2 were selected for performing the task as these pre-trained models has proven benchmark results in terms of improved recognition accuracy using minimal scaling factors (light weight model).

**Fig. 5:**
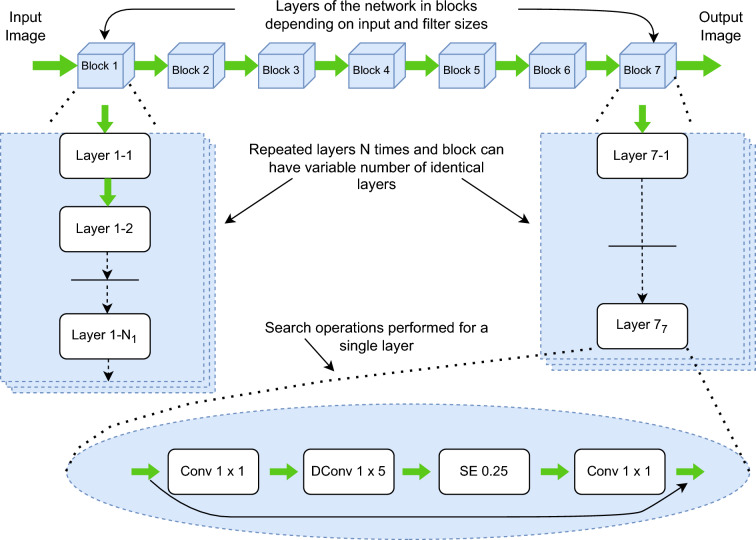
Visual representation of NASNet Mobile architecture^32^.

**Fig. 6:**
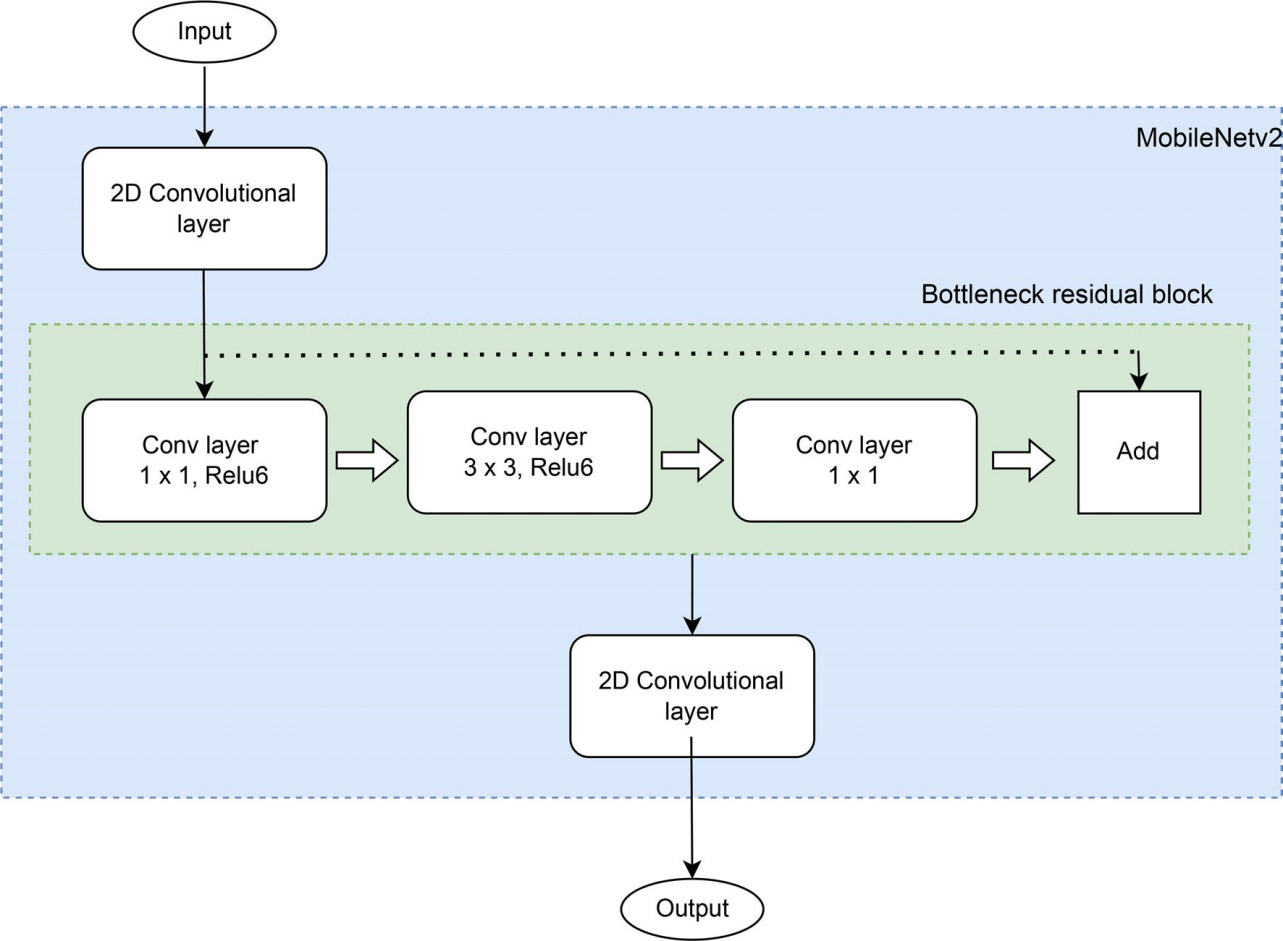
Visual representation of MobileNetV2 architecture^33^.

#### NASNetMobile architecture

The neural architectural search (NAS) is a novel algorithm that helps to design the most suitable architecture and model for a particular dataset, in terms of minimal scaling and increase in accuracy. NAS technology helps to design a neural network architecture suitable for a particular problem or use-cases in a faster and better manner automatically^32^. This system consists of four main building blocks: search space, use-case, search strategy or model generator, algorithms and model evaluation. It is flexible for hyperparameter tuning and determine the optimal structure of the learning model that provides better performance than other hand coded algorithms. More than a million images from the benchmarked CIFAR-10 dataset and ImageNet database were used to train a CNN known as NASNet-Mobile. Figure 5 shows a visual representation of the NASNetMobile architecture.

#### MobileNetV2 architecture

MobileNetV2 CNN was designed to adapt and perform well on mobile devices. The core idea of MobileNet version 1 is the replacement of expensive convolutions with light weight blocks. This method works based on an inverted residual framework, where residual connections exist between the layers that are bottle-necked^33^. Figure 6 shows a visual representation of the MobileNetV2 architecture. The intermediate expansion layer extracts non-linearity from the features by filtering them using lightweight depthwise convolutions. Overall, the MobileNetV2 architecture consists of a fully connected convolutional layer containing 32 filters at the beginning and 19 residual bottleneck layers. The MobileNetV2 has better performance than other recent CNN architectures, even though it uses 30% fewer parameters and minimum CPU runtime. Hence, this version was chosen to meet the needs of the application and dataset.

### Proposed method and its significance- mutated ensemble learning with NAS optimized attention

In this study, an ensemble system outperforming transfer learning (TL) models is proposed for diagnosing micro nutrient deficiency in banana crops. Our proposed approach consists of three stages: (1) mutation in transfer learning, (2) ensemble of the mutated models and (3) NAS Optimization. To further enhance diagnosis, the study introduces the quantification of the severity levels using Greenness Index (GI) value and represent them as three levels mild, moderate and severe. The proposed system integrates NASNetMobile and MobileNetV2 in an attention-based ensemble, where each model analyzes the input image and a Dense Softmax layer assigns trainable attention weights. The final decision is made via a weighted sum of outputs, ensuring adaptive contribution of each base model. Training is performed with categorical cross-entropy loss and optimized using the Adagrad optimizer. This combination of architecture optimization, adaptive weighting, and severity quantification ensures robust performance across accuracy, precision, and recall metrics.

#### Mutation in transfer learning

A pre-processed image with dimensions of 224 × 224 was fed into the mutated pre-trained neural network. To carry out the objective, initially the neural layers of base learners are modified (which we call mutation) so that a specific dataset can be scaled and classification operations can be performed as explained below. The TL models were modified by replacing the top layer with two dense layers, globalaveragepooling2D, with a dropout of 0.5 to avoid overfitting and was evaluated with the selected banana dataset collected from Mendeley. The training was performed with 30 epochs, keeping the batch size at 16, learning rate at 0.01857 and callbacks such as reducing learning rate and early stopping technique was used to decide the best overall weights and training was stopped if there was no improvement in validation accuracy up to five epochs (patience=5). The evaluation parameters and layer descriptions of the selected TL models are listed in Tables 4 and 5 respectively.

**Table 4:** Evaluation parameters of mutated transfer learning models.

Models	Total parameters	Trainable parameters	Non-trainable parameters
Mutated NASNetMobile	4,607,128	337,412	4,269,716
Mutated MobileNetV2	2,652,740	394,756	2,257,984

**Table 5:** Layer description in mutated models.

Layer type	Mutated NASNetMobile		Mutated MobileNetV2	
	Output shape	Total parameters	Output shape	Total parameters
Input layer	(None, 224, 224,3)	0	(None, 224, 224,3)	0
NASNetMobile	(None, 7, 7, 1056)	42,69,716	(None, 7, 7, 1280)	22,57,984
Global Averagepooling2D	(None, 1056)	0	(None, 1280)	0
dense1	(None, 256)	2,70,592	(None, 256)	3,27,936
dense2	(None, 256)	65,792	(None, 256)	65,792
dropout	(None, 256)	0	(None, 256)	0
Activation	(None, 4)	1028	(None, 4)	1028

#### Ensembling the mutation

Algorithms for ensemble learning operate by combining different base learners and creating hypotheses in response to the results. There are various methods of ensembling: Bagging, Boosting, Voting, Stacking,ensemble averaging and weighted ensemble learning. The ensemble averaging approach calculates the estimated probability of multiple base models, whereas the weighted ensemble learning approach merges model outcomes and algorithms by voting procedure in order to select the outperforming model. In this study, after the experimental training and validation results of the mutated TL models, ensemble classifier was formed using simple averaging strategy based on the accuracy and loss parameters. The study integrates modified versions of NASNetMobile and MobileNetV2 models. The layers with their output shapes and parameter details in the structured layers are listed in Table 6.

**Table 6:** Layers and parameters of ensemble classifier- NasMobv2.

Layer	Output shape	Total parameters
Input layer	(None, 224, 224,3)	0
Functional	(None, 4)	4607128
Functional	(None, 4)	2652740
Average	(None, 4)	0

The ensemble learning parameters for training are as follows:- Total parameters: 7,259,868; Trainable params: 732,168; Non-trainable params: 6,527,700. The ensemble based algorithm is shown in Algorithm 1,

**Algorithm 1:**
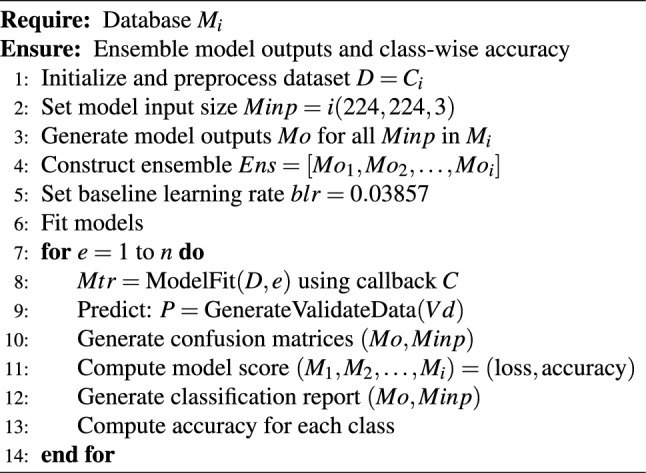
Averaging Ensemble Strategy

where $M_i$ is the total number of models selected for evaluation, D=dataset, Ci=the number of classes, blr is the base learning rate,e is the number of epochs,i is the input shape of the image, Mc is the current model fed with input into a set of models (Mi). The ensemble (Ens) model is created using Mo1,...Moi by comparing it with performance metrics. Plot the Accuracy curves with epoch(along the Y-axis) and accuracy(along the X-axis), similarly plot loss curves = epoch(along the X-axis) and loss(along the Y-axis). As the above algorithm is the base and in order to optimize the classification focusing on features that enables the classification, which is explained in the following subsection.

#### NAS-optimized attention based ensemble

The Proposed Model combines NAS with an attention-based ensemble of pre-trained models such as NASNetMobile and MobileNetV2, to improve the classification of nutrient deficiencies.

**Fig. 7:**
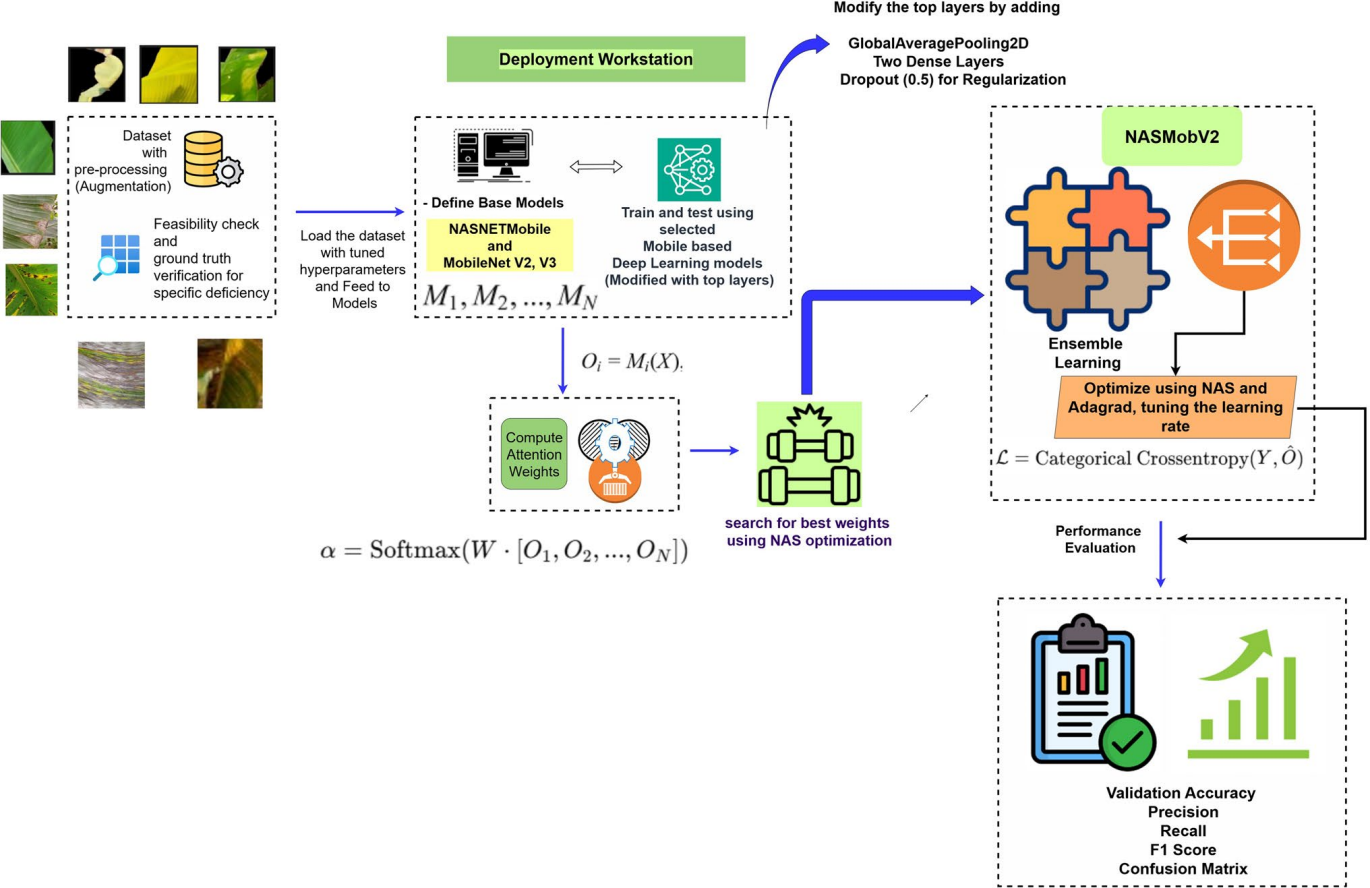
Proposed methodology - **NasMobV2** (Mutated- NASNETmobile + MobileNetV2).

Every model analyzes an input image, and a Dense Softmax layer refines the output by allocating trainable attention weights to dynamically calculate the contribution of each model. Following training with categorical cross-entropy loss and refinement using the Adagrad optimizer, the final prediction was derived by computing the weighted sum of the model outputs. By utilizing both architecture optimization and adaptive weighting techniques, the model ensures robust performance when assessed in terms of accuracy, precision, and recall. The Algorithm 2 used in the NAS driven ensemble is shown below. 

**Algorithm 2:**
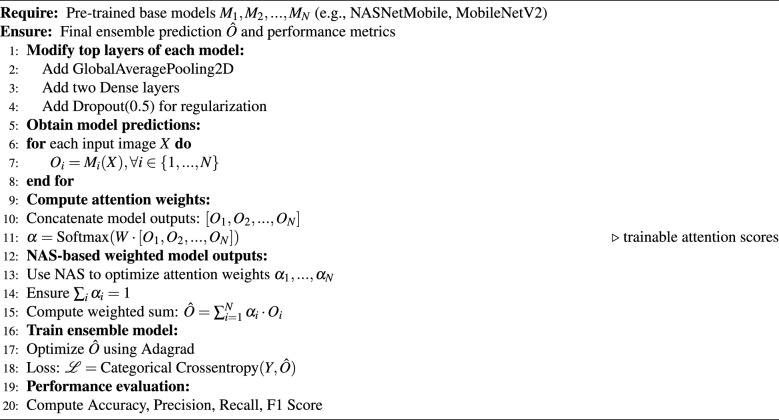
NAS-optimized attention-based ensemble model for nutrient deficiency classification

### Mathematical notations

- *Input and output*
  - $X \in \mathbb {R}^{H \times W \times C}$ : input image, where *H* = height, *W* = width, *C* = number of channels.
  - $Y \in \{0,1\}^K$ : one-hot encoded ground-truth label for *K* classes.
  - $\hat{O} \in [0,1]^K$ : final ensemble output probability vector over *K* classes.
- *Base models*
  - $M_i$ : *i*-th pre-trained base model ($i \in \{1,2,\dots ,N\}$), e.g., NASNetMobile, MobileNetV2.
  - $O_i = M_i(X) \in [0,1]^K$ : predicted probability distribution of class labels by model $M_i$.
- *Attention weights*
  - $[O_1, O_2, \dots , O_N]$ : concatenated prediction vector of all models.
  - $W \in \mathbb {R}^{K \times NK}$ : trainable weight matrix for attention mechanism.
  - $\alpha = \text {Softmax}\big (W \cdot [O_1, O_2, \dots , O_N]\big )$ : normalized attention weight vector.
  - $$\alpha = [\alpha _1, \alpha _2, \dots , \alpha _N]$$, with $$\sum _{i=1}^N \alpha _i = 1$$ and $$\alpha _i \ge 0$$.
- *Final ensemble prediction*
  - $\hat{O} = \sum _{i=1}^{N} \alpha _i \cdot O_i$ : weighted sum of model predictions.
- *Loss function*
  - $\mathcal {L} = - \sum _{k=1}^K Y_k \log (\hat{O}_k)$ : categorical cross-entropy loss, where $Y_k$ and $\hat{O}_k$ denote ground-truth and predicted probability for class *k*.

The approach contributes to optimizing the ensemble weights between two pre-trained models, NASNetMobile and MobileNetV2. After constructing an input layer (224 × 224 × 3), trainable weights (weight1 and weight2) are assigned, ensuring that their sum is equal to 1. The output of the contribution of each model to the ensemble was handled using these weights. Using a Lambda layer, a weighted sum operation was used to calculate the combined result. The model is also assembled using the Adagrad optimizer, wherein the learning rate can be varied using values (0.018750, 0.01750, 0.002). A Bayesian Optimization-based neural architecture search (NAS) using Keras Tuner was implemented to optimize the ensemble weights and learning rate. The Fig. 7 shows the proposed methodology. The goal is to obtain the best classification performance with important metrics such as accuracy and categorical cross-entropy loss. The tuner was set up to improve the validation accuracy across ten trials. Ten epochs of search were conducted using the dataset.

### Deployment of model as web and mobile app

After experimentation and performance analysis, the developed ensemble model was deployed as a web and mobile application to observe how the model executes robust data and to view its capabilities using the streamlit web tool and Android studio after converting the saved ensemble model to the TFlite model. The input images were classified as expected and a confidence score for the classification was displayed.

#### Web application deployment

**Fig. 8:**
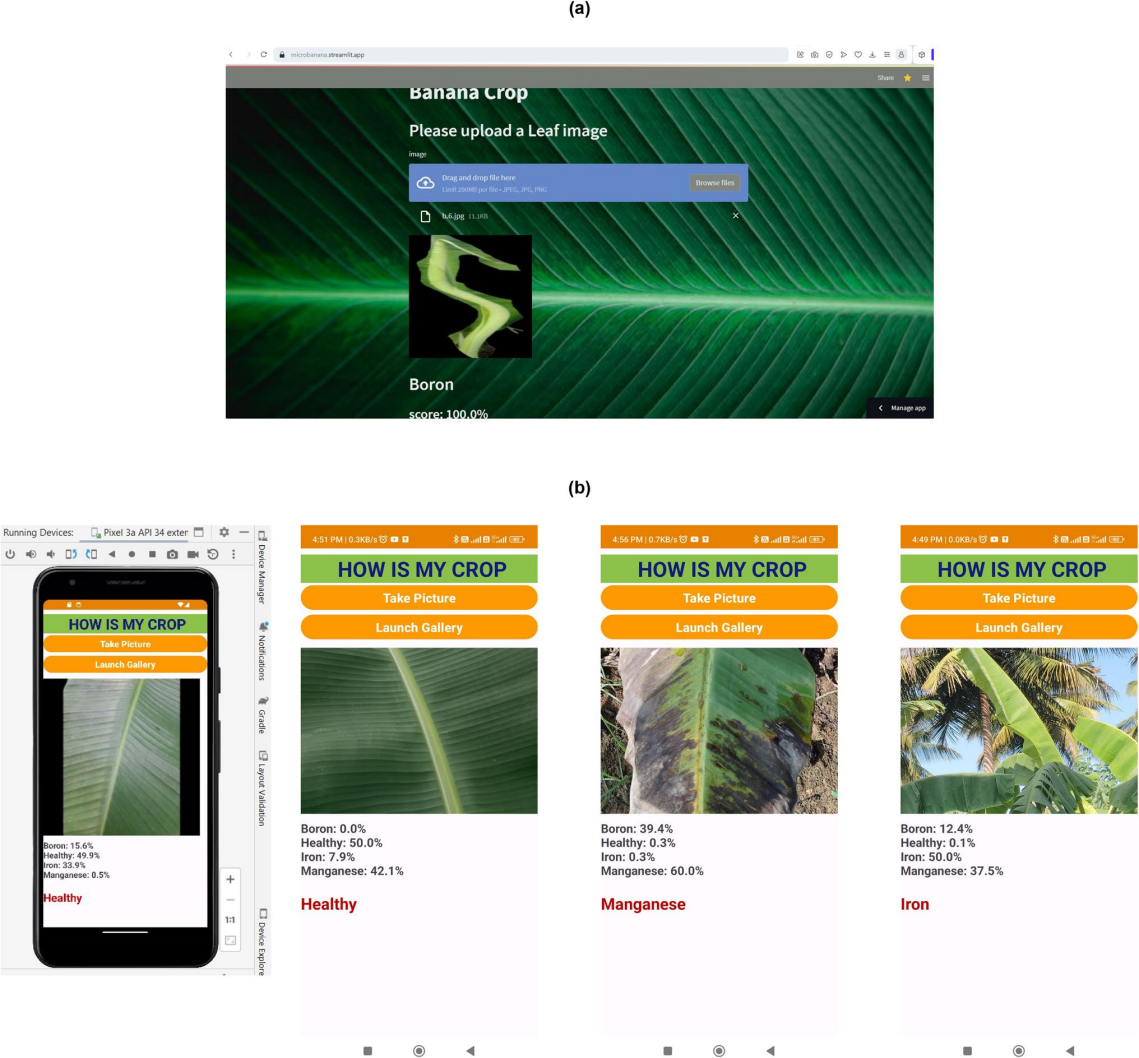
(**a**) Web application; (**b**) Mobile application and sample implementations.

The streamlit tool for creating a web application was used to convert the saved hd5 model into a streamlit application and an interface was created using the html file. The web application titled “Banana crop deficiency classification” was tested for real time captured images and other test images of banana leaves with micro nutrient deficiency as well as healthy images. Figure 8a shows a view of the implemented web application, where the user is expected to upload a banana leaf image using a browse button or drag the file in the generated upload bar. After the file is uploaded, the model is executed for classification and the class results are shown below along with the confidence score.

#### Mobile application deployment

Android Studio giraffe version 2022 was used for the emulating. Here, The ensemble model was converted into a tflite format for integration with the development of mobile application and tested with the images. We call the application as “How is my crop”. The sdk version was set to the maximum of APi-34 and was initially tested with the pixel -3a emulator. Later, the application was converted to the apk format and tested with mobile models such as Redmi 3s- prime and Redmi 12 - 5G. The app size was 32.5 MB. Figure 8b depicts a screenshot of the mobile application created for the banana micro-deficiency classification. The classification was carried out for banana crop leaves by examining the confidence scores of the selected classes and the final result was obtained. The application resulted in image classification for selected micro deficiencies or healthy leaves. The converted TFLite model reported the following statistics within JupyterLab: Model size: 28.88 MB (28884192 bytes) Non-data buffer size: 266.6 KB (0.92%) Data buffer size: 28.61 MB (99.08%) Zero-value buffers: 1.5 KB (0.01%). To evaluate deployment feasibility, we benchmarked the model on-device: Inference time:  92 ms (Pixel-3a),  63 ms (Redmi 12 5G) per image (224$\times$224 input). Memory footprint: Peak RAM usage  112 MB during inference. Battery consumption: Continuous inference for 100 images led to  2.8% battery drop on Redmi 12 5G (5000 mAh), suggesting low power overhead. These results confirm that the proposed model is lightweight and suitable for real-time mobile deployment in agricultural field conditions.

### Nutrient level quantification

The identification and calculation of leaf deficiency severity levels in the crop leaves are explained in this section. Concurrent nutrient stresses may arise in plants in real-time environment. In order to identify this multi-nutrient stress, we tend to quantify the level of identified nutrients to find the severity. The green colour of the leaves is indicative of the general health of the plant, which indicates the health status of the plants. The Green Index (GI) is used to evaluate the levels of multi-nutrient stress in plant leaves. It is imperative to recognise that there is a distinct correlation between the Chlorophyll Metre Index (CMI) value and the GI value^34^. The degree of nutrient stress in plant leaves is indicated by this CMI value. It is necessary to perform this computation using the following steps: The leaf dataset is gathered and after pre-processing such as resizing, scaling, augmentation, etc,the images are converted to a standardized RGB color space. Feature extraction was carried out to capture the greenness values of the leaves using the green channel intensity values from the RGB color space. To describe the greenness of each leaf image, compute metrics such as mean greenness, median greenness, or greenness distribution histograms,which are described in detail below.

#### Steps for calculation of severity in m-ND

Several sequential steps are involved in determining the Greenness Index (GI) value. The first step involved splitting plant leaf images into three different color channels: red, green, and blue. Individual processing was then performed for each color channel. The GI value was calculated using the following equation,

1 $$\begin{aligned} G I=\frac{G}{R+G+B+1 e-6} \end{aligned}$$

where R is the normalized red channel intensity and G is the normalized green channel intensity. The Degree of Severity (DoS) for severity was calculated as follows:

- Image Pre-processing: Images from the dataset were converted into the RGB color space.
- Partitioning leaves: Leaves can be separated from the background using image segmentation techniques. Among the various segmentation approaches, we used thresholding based on deep learning and morphological operations to remove noise.
- Finding the Symptoms: Locating any leaf regions that are symptomatic. These patches can have different textures or colour changes (e.g., yellowing). This can be accomplished through other image analysis techniques, such as color thresholding or machine learning. Initially, the image was converted to HSV space and the yellow coloring was estimated using the standard ranges between lower yellow and upper yellow.
- Classifying Severity: The degree of deficiency was assessed by calculating the ratio between the symptomatic area and the total leaf area.

**Fig. 9:**
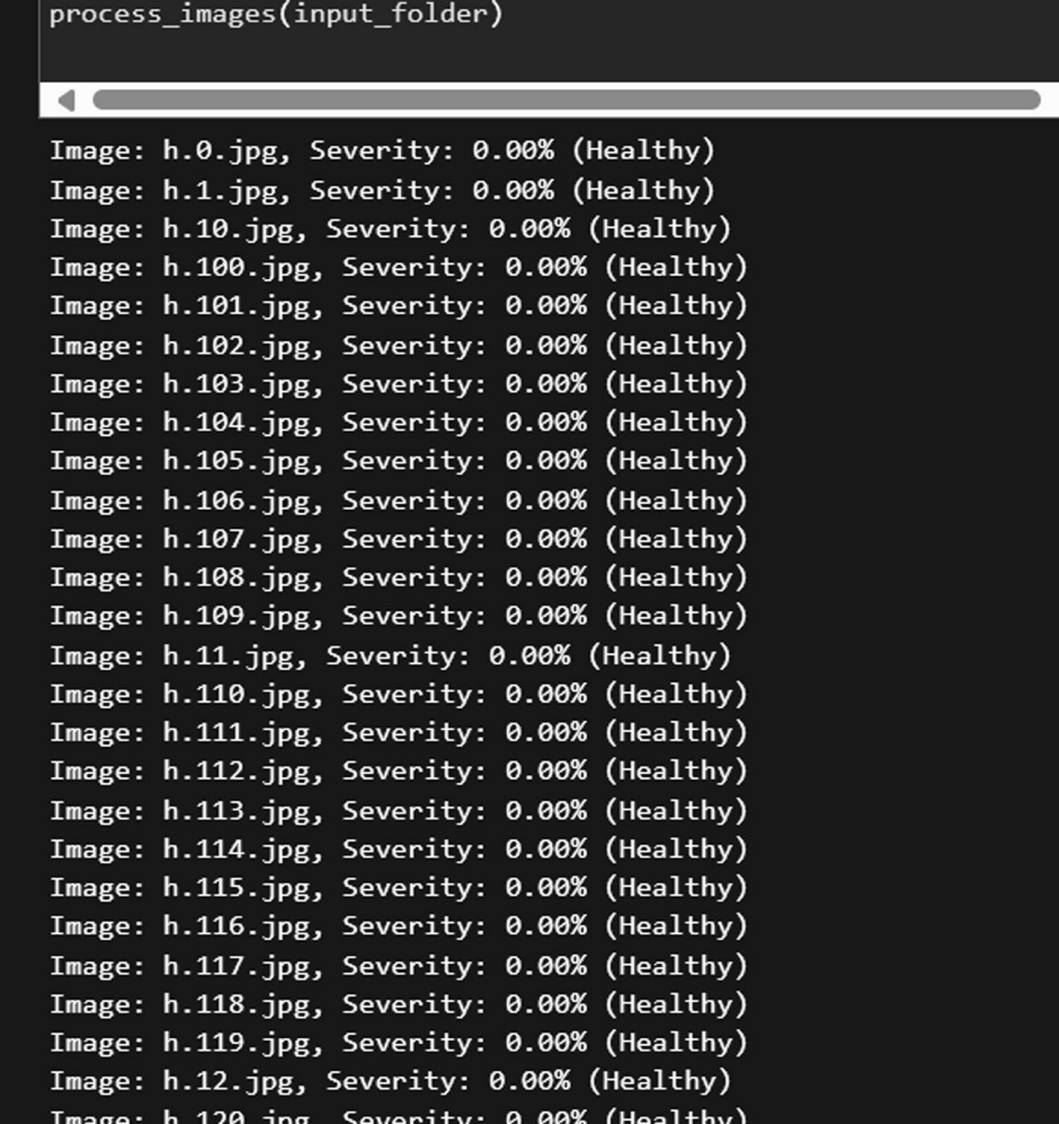
Sample results for estimation of severity.

To further classify the severity accuracy, the mean value is calculated, focusing on the green channel in particular, and then used this mean value to find the range of the lowest and maximum pixel values. For instance, if the mean value of green channel is denoted by X, then X is assigned to the channel’s initial range of pixel values. Next, the likelihood (P) of crossing X within the green channel is calculated. The intensity value range, from minimum to maximum, gradually increased by one unit in cases when the calculated probability (P) was less than 0.9. The iterative process continued until P reaches a preset threshold of 0.9. This methodology is used to effectively remove abnormally low and high intensity values from the image channel, thereby augmenting the accuracy of GI as a plant health indicator. The Fig. 9 depicts the sample estimation results of the severity classification.

Any further increases to the minimum and maximum ranges were stopped once the P value reaches the 0.9 threshold. The Green Channel Value (GCV) is then computed by adding up all the intensity values of the image pixels and dividing that total by the number of pixel values in the channel. The same process is repeated for the red and blue channels of the input image. A GI value above 1.05 indicates a high degree of nutrient deficiency in the leaves, while a score below 0.95 indicates a minor degree of nutrient deficiency. On the other hand, when the GI score falls between 0.95 and 1.05, significant nutritional deficiency is not present.

### Performance analysis

In general, a ML/DL model has been evaluated for its performance using various parameters and metrics^35^. The proposed model is examined by comparing a number of metrics, including accuracy, precision, recall, and F1 score, and by utilizing the confusion matrix. Their equations are expressed below as Eqs. (2), (3), (4) and (5), where: A denotes the true positive; B the true negative; C the false positive; and D the false negative.

2 $$\begin{aligned} & Accuracy =\frac{\sum A+\sum B}{ \sum A+\sum B+\sum C+\sum D} * 100 \end{aligned}$$

3 $$\begin{aligned} & Precision =\frac{\sum A}{\sum A+\sum C} * 100 \end{aligned}$$

4 $$\begin{aligned} & Recall =\frac{\sum A}{\sum A +\sum D} \end{aligned}$$

5 $$\begin{aligned} & F1\hspace{0.1 cm}score = 2 *\left( \frac{{ Precision } * { Recall }}{{ Precision }+ { Recall }}\right) * 100 \end{aligned}$$

## Experimental results and discussion

In this study, the validation accuracy was used to compare the rank in the prediction and test results of two light weight CNN models. The primary aim of this study was to identify and compare the performance of ensemble learning with that of individual learners. Initially, training were performed using pre-trained CNN models such as NASNetMobile, MobileNetV2 and MobileNetV3 with the modified top classification layers. The prediction performance of Mobilenet version3 was not satisfactory compared to the selected models. It was closer to NASNetMobile, but less than that of MobileNetV2. Hence, the advanced version of MobileNet is less accurate in terms of our objective. Therefore, MobileNetV3 was ignored. Because the selected models are light weight and combined with the ensembling methodology, the training time is less than that of regular CNN models. Table 7 presents the results of the different regular CNN models.

**Table 7:** Comparison of the results obtained for various regular CNN models(4 classes).

Model	Validation accuracy (%)	Validation loss (%)	Precision	Recall	F1-Score
ResNet50	62.50	23.15	61.50	63.60	62.53
RegNetY002	94.50	23.12	95.21	94.37	94.79
VGG16	88.00	32.00	87.10	88.60	87.84
EfficientNetV2B1	88.37	32.63	90.41	87.25	88.80

### Transfer learning

The lightweight TL models namely NASNetMobile, MobileNetV2 was selected for training and validation. However the validation loss and accuracy were initially not improved, hence tuned the hyperparameters such as varying the batch size, epoch count and learning rate. The models performed better after the tuning. The accuracy scores for the models was ranging from 95 to 96.38%. The categorical scores and accuracies for each category are listed in the Tables 8 and 9 respectively. The number of actual values (ground truth) for Boron, Healthy, Iron and manganese were 81, 69, 72 and 83 respectively. In terms of individual classifiers,the MobileNetV2 model provided the highest test accuracy compared to MobileNetV1 or V3 and NASNetMobile.

**Table 8:** Categorical prediction parameters of various TL models(4 classes).

Models	Deficiency prediction parameters															
	Boron				Healthy				Iron				Manganese			
	P	R	F1	S	P	R	F1	S	P	R	F1	S	P	R	F1	S
Mutated NASNetMobile	92	94	93	81	94	99	96	69	71	96	82	72	96	61	75	83
Mutated MobileNetV2	99	95	97	81	99	97	98	69	71	97	82	72	95	70	81	83
Mutated MobileNetV3	91	93	95	81	94	98	96	69	71	94	80	72	94	60	73	83

**Table 9:** Accuracy of each category (4 classes).

Model	Boron (%)	Healthy (%)	Iron (%)	Manganese (%)
Mutated NASNetMobile	93.82	98.55	95.83	61.44
Mutated MobileNetV2	95.06	97.10	97.22	69.87
Mutated MobileNetV3	92.6	98.6	94.4	60.2

### Ensemble models

**Fig. 10:**
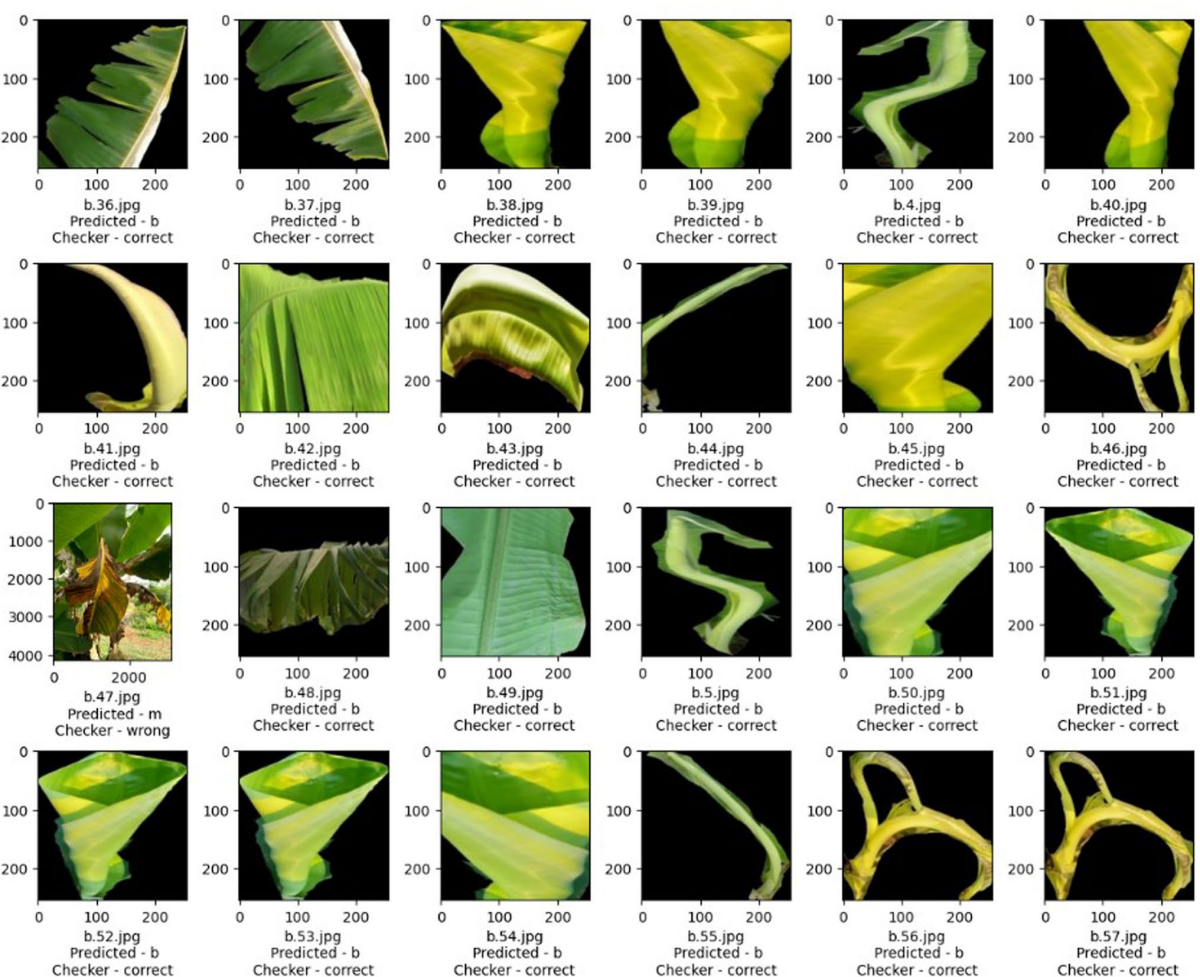
Sample predicted results.

**Fig. 11:**
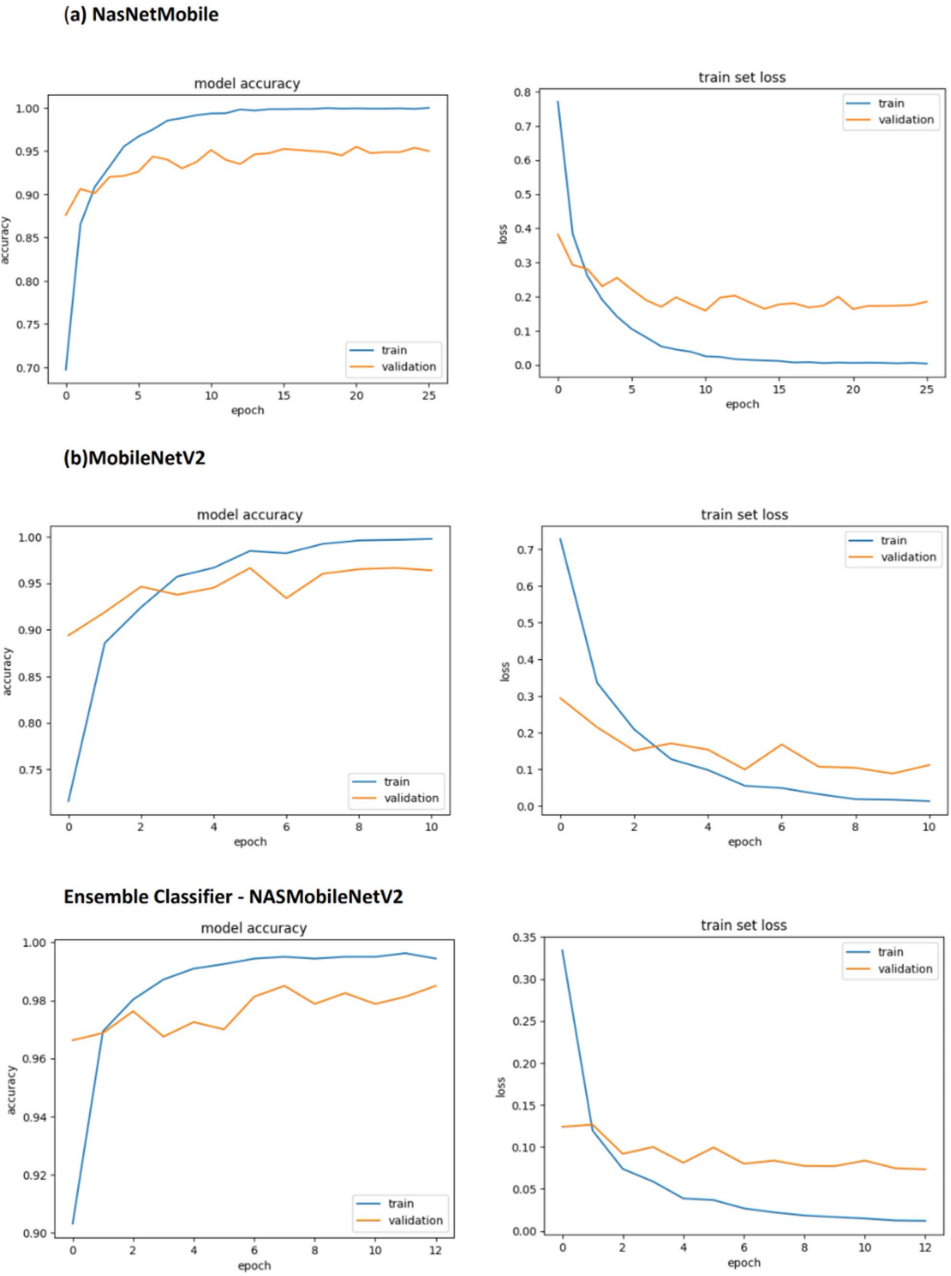
Accuracy and loss curves.

**Fig. 12:**
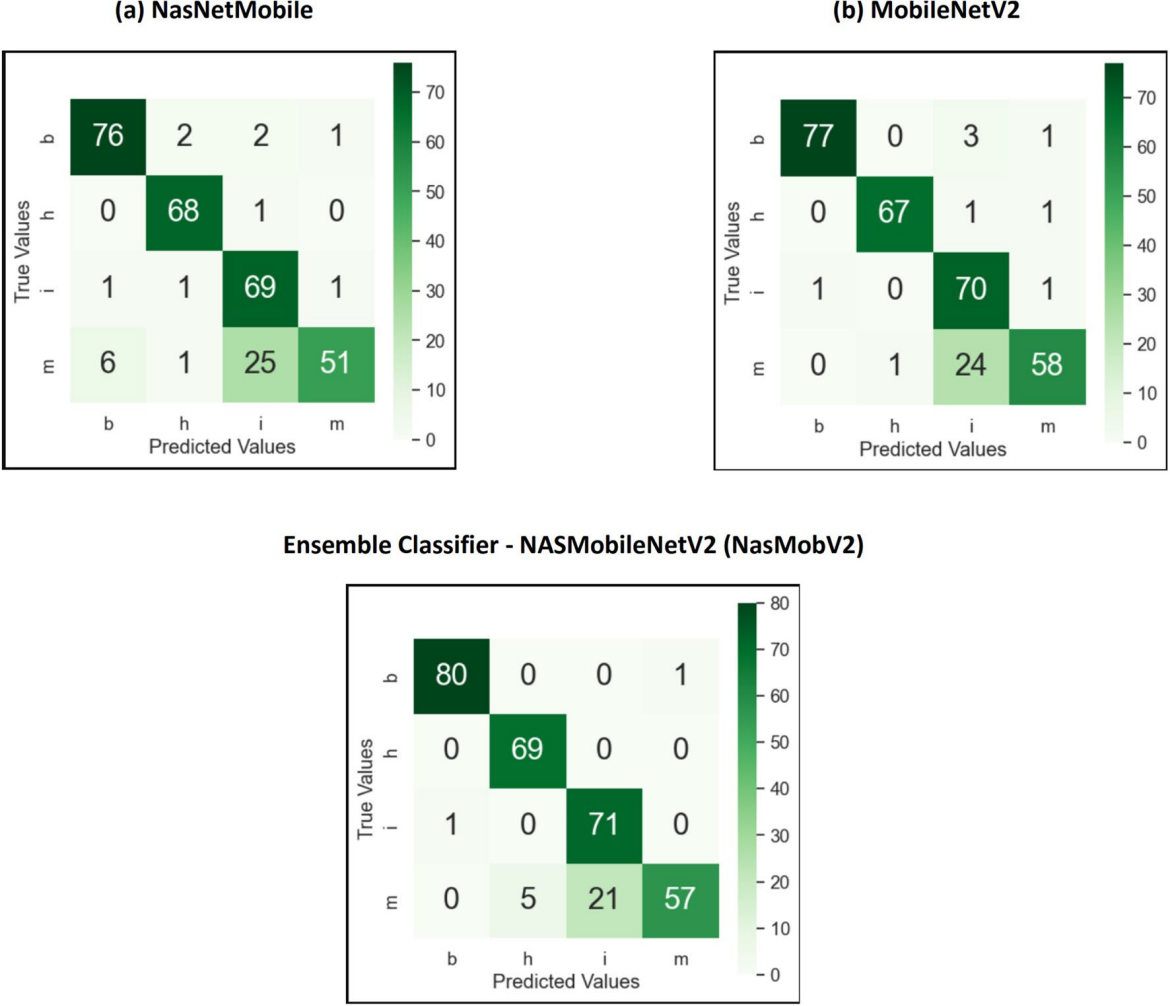
Confusion matrices.

After evaluating the metrics, confusion matrix and classification report for TL models, the ensemble classifier was built using the prediction performance of both models MobileNetV2 and NasNetmobile, which tends to improve the classification accuracy and minimize the loss or wrong predictions. The individual and ensembled classifiers were tested using 250 images. Initial performance results were obtained using simple averaging ensembling strategy, accuracy of 97.8%. As this method leverages the models without giving specific priority to a better-performing model, experimentation with the NAS driven optimization was done. The NAS-optimized ensemble classifier NASMobileNetV2 **(NASMobv2)** had a good performance on the banana micro nutrient deficiency datatset attaining overall accuracy of 98.57% and a f1 score of 91%. The overall performance of the various models is tabulated in Table 10 and Fig. 10 shows the sample prediction results. Figures 11 and 12 shows the Accuracy, Loss curves and confusion matrices respectively. The performance metrics for the coffee dataset is shown in Table 11.

**Table 10:** Overall performance of models (4 classes).

Model	Precision (%)	Recall (%)	F1 score (%)	Accuracy (%)
Mutated NASNetMobile	95.23	94.87	86	95.00
Mutated MobileNetV2	96.50	96.38	89	96.38
Mutated MobileNetV3	95.37	94.77	85	95.12
**NAS-Optimized NASMobv2(Proposed)**	**98.62**	**98.25**	**91**	**98.57**

**Table 11:** Performance metrics for coffee leaf (Co-Leaf DB) dataset.

Proposed model/Metrics –>	Accuracy (%)	Precision (%)	Recall (%)	AUC (%)	F1 Score (%)
NASMobV2 (Proposed)	95.2	95	92	95	95

**Table 12:** Comparative evaluation of mutated transfer learning models and proposed ensemble in terms of model parameters, approximate FLOPs, and inference times.

Model	Params (M)	FLOPs (M)	GPU (ms)	CPU (ms)
Mutated NASNetMobile	4.61	$\sim$550 to 600	$\sim$5 to 7	$\sim$40 to 50
Mutated MobileNetV2	2.65	$\sim$280 to 320	$\sim$3 to 4	$\sim$20 to 25
Mutated MobileNetV3	22.39	$\sim$800 to 1000	$\sim$8 to 12	$\sim$60 to 80
Proposed Ensemble (NASMobv2)	7.26	$\sim$850 to 900	$\sim$7 to 10	$\sim$50 to60

**Table 13:** Per-class classification metrics of the proposed model for nine classes.

Class	Precision	Recall	F1-Score	Support
Boron	0.980	0.976	0.978	250
Healthy	0.972	0.980	0.976	250
Iron	0.984	0.984	0.984	250
Manganese	0.996	0.980	0.988	250
Cordana	0.972	0.984	0.978	250
Pestalotiopsis	0.976	0.984	0.980	250
Sigatoka	0.984	0.988	0.986	250
Zinc	0.996	0.992	0.994	250
Potassium	0.996	0.988	0.992	250

**Fig. 13:**
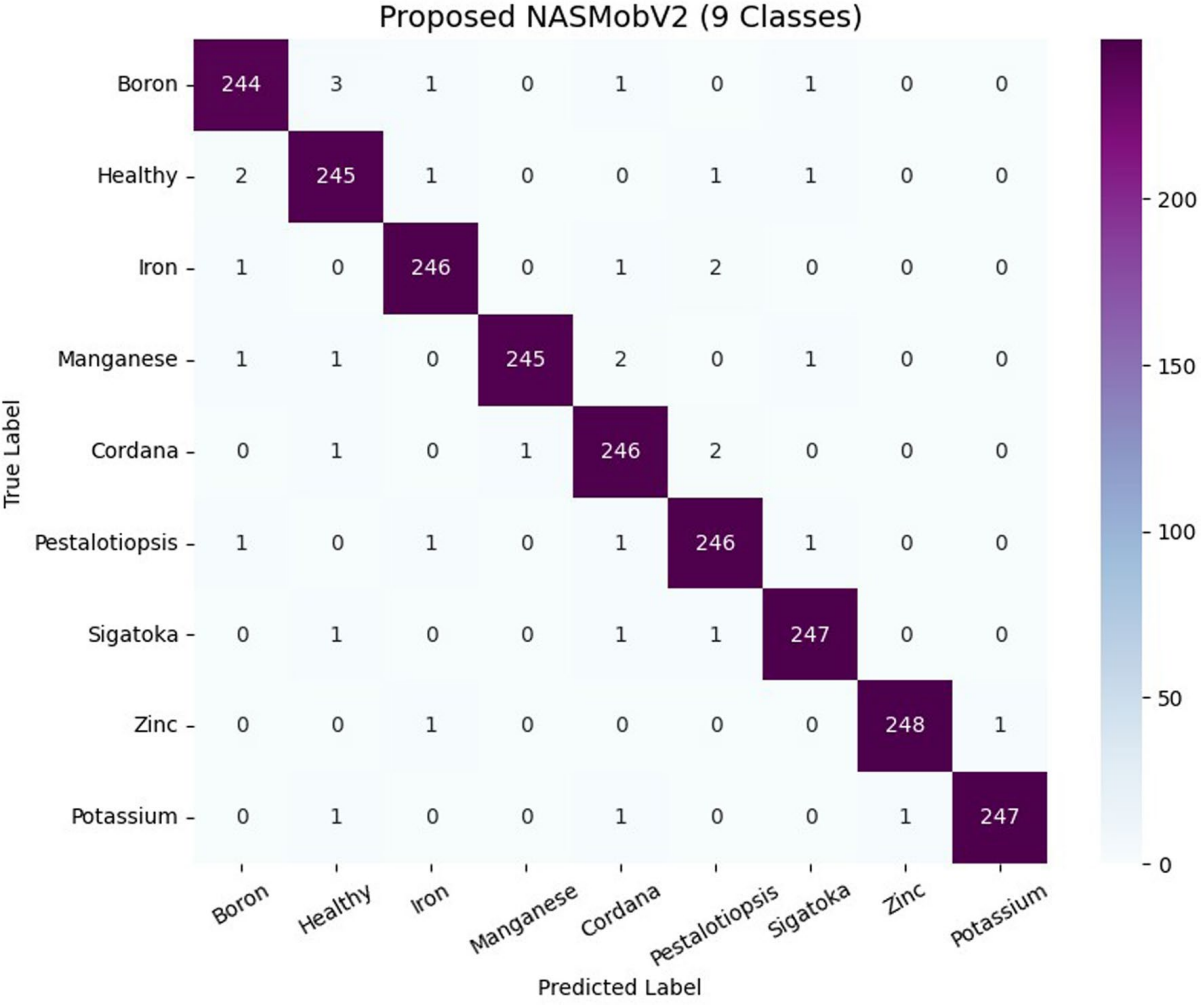
Confusion matrix for 9 classes of banana dataset.

**Fig. 14:**
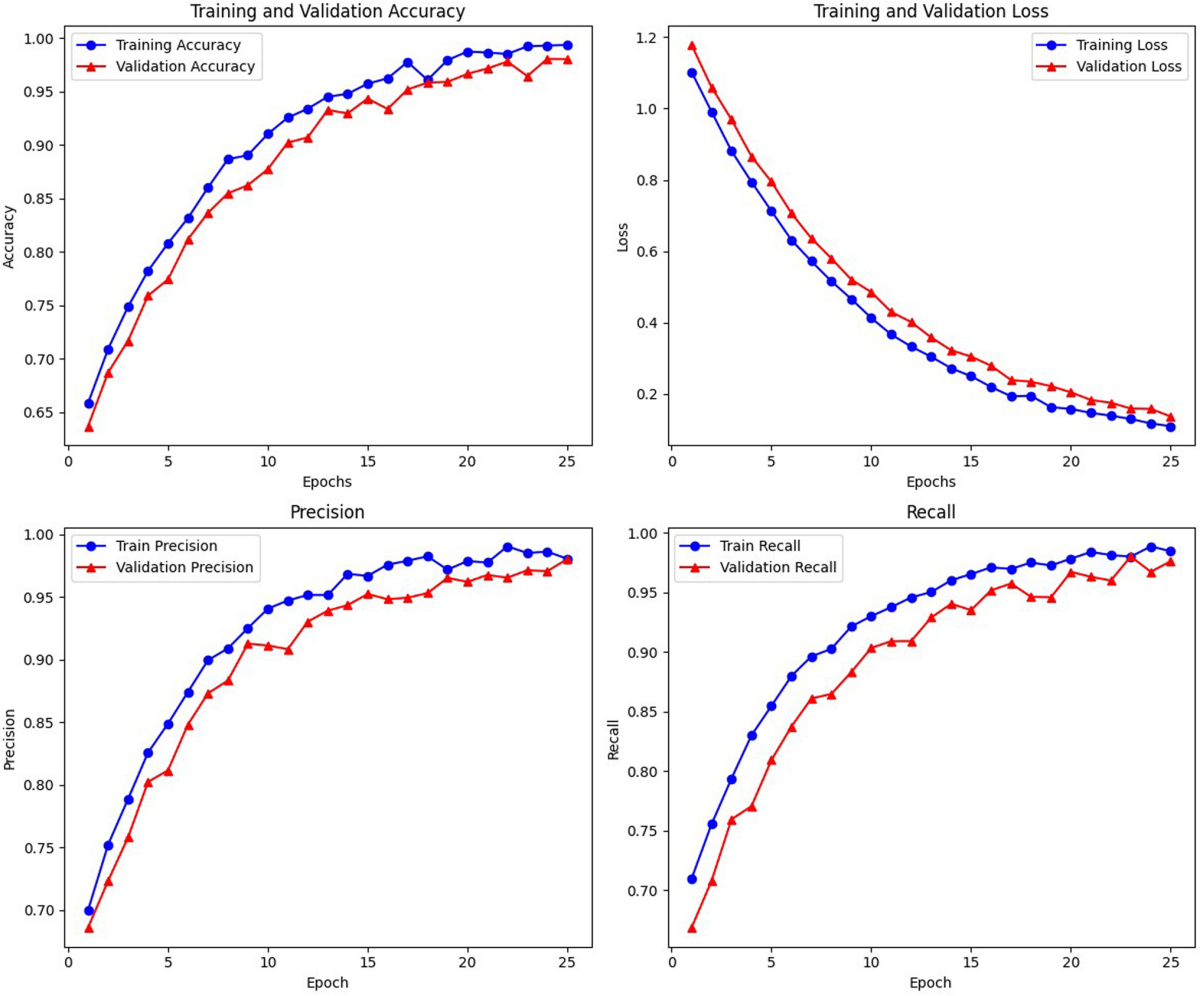
Accuracy and loss curves for Nine classes of banana dataset.

**Fig. 15:**
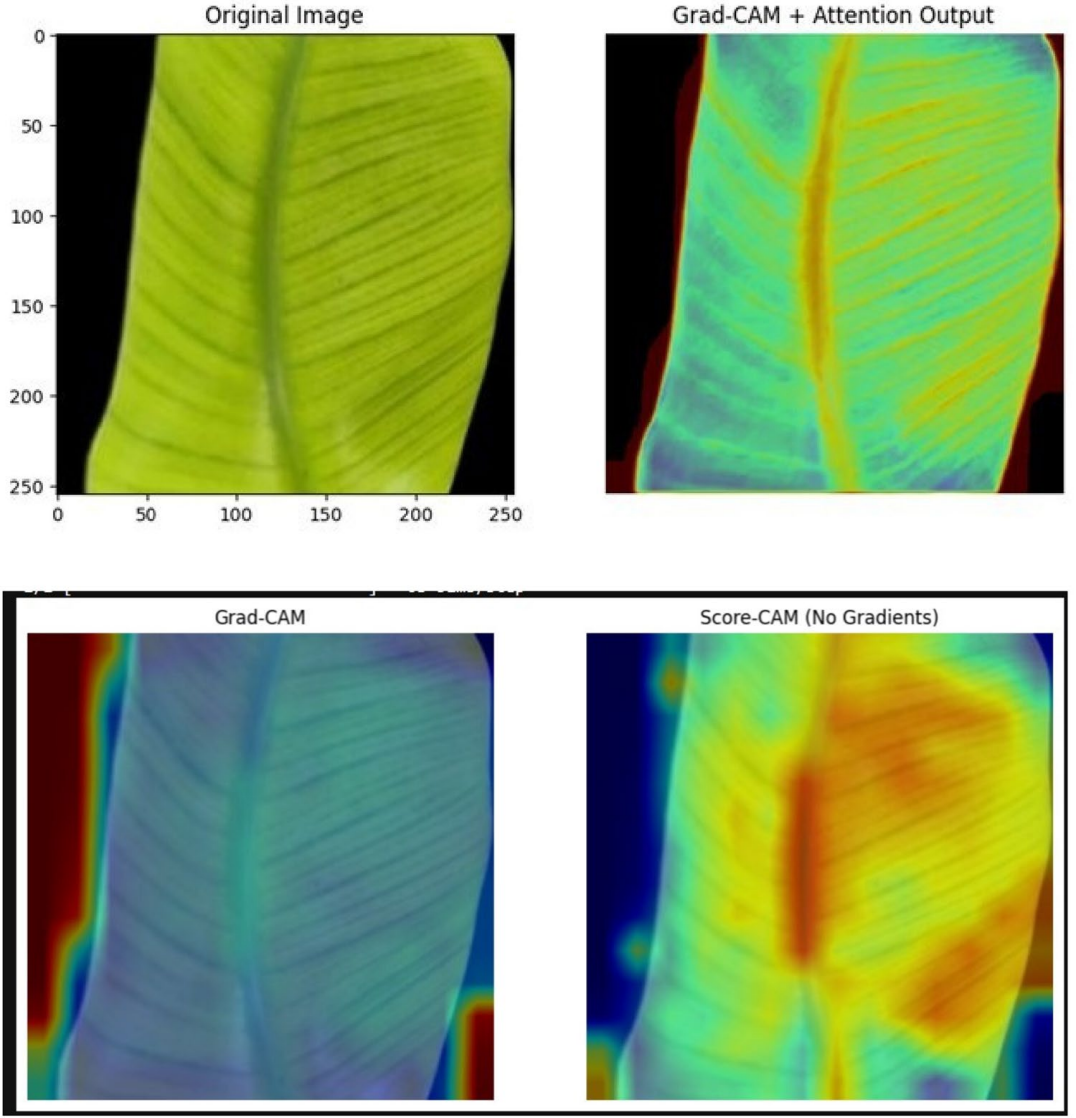
Visualization of model classification using Grad-CAM, Score-CAM and Attention.

### Discussion

Initially, various original CNN models including ResNet50, VGG16, and EfficientNet were trained. However, the above original CNN models performed poorly for our selected dataset with regard to the validation accuracy and the parameters were large enough to train, which leads to time complexity constraints. The ResNet50 achieved 62.50% validation accuracy and loss of 23.15%, RegNetY002 achieved 94.50% accuracy but the loss was 23.12%, VGG16 achieved 88.10% accuracy and loss was of 32%. The timing constraint ranged from approximately 250 to 510 s per epoch. Therefore, mutation was attempted at the classification layers, such as setting the two dense layers (None,256) combining with the Adagrad optimizer and callbacks with epoch patience of 5. The base learning rate is 0.01857. The total epoch size tended to be 32–40.

The assessment of the selected TL models and the proposed ensemble model was performed, based on performance metrics such as precision, recall, F1 score, support and accuracy for the labelled classes or categories. During the experimental trials and hyperparameter tuning, the best models were selected which performed better in terms of both training and validation. The suggested model attained the following true predictions: 80 out of on 81 for boron, 69 out of 69 healthy, 71 out of 72 iron and 57 out of 83 manganese categories. Figure 13 represents the confusion matrix of the proposed model for the expanded dataset of nine classes of deficiency and diseases in banana crop. Table 12 depicts the comparison between the various baseline models and proposed in terms of Parameters, FLOPS, GPU, CPU utilizations. Although direct energy consumption measurements (e.g., Joules/Watt) were not performed, The model size, FLOPs, and inference times were reported as widely used proxy indicators of computational efficiency. Compared to standard NAS baselines such as NASNet-A Large (88M parameters, $\sim$23B FLOPs, $\sim$350 MB model size),the proposed ensemble achieves an order of magnitude reduction in model size (27.7 MB vs. 350 MB) and significantly fewer FLOPs ($\sim$0.9B vs. 23B), indicating lower memory footprint and reduced computational overhead. While the mutated MobileNetV3 variant is heavier (22M parameters), it was included for ablation and comparison, whereas the ensemble of NASNetMobile and MobileNetV2 represents the optimal trade-off between accuracy and efficiency. The per class classification metrics is tabulated as Table 13.

Hence, the proposed model not only improves predictive performance but also offers a computationally lighter alternative to conventional NAS baselines, supporting its suitability for deployment in real-time agricultural applications. With regard to the practical implications, Though the proposed model achieves high accuracy on benchmark datasets, real-world conditions such as leaf occlusion, overlapping foliage, heavy shadows, and background clutter may reduce performance. This limitation is common in current image-based plant diagnostic systems. To partly address these challenges, our training pipeline included augmentations (random cropping, brightness/contrast variations, and cutout) to simulate occlusion and shadow conditions. The performance graphs such as accuracy and loss curves for nine classes of deficiencies with diseases are depicted in Fig. 14. As our next research direction and work, we plan to augment training data with synthetic occlusion/shadowing, employ self-supervised pretraining to handle noisy field images, and explore transformer-based segmentation to better isolate leaf regions under complex backgrounds.

#### Comparison of one step strategy vs proposed two stage strategy

In this study, a systematic comparison is performed for the evaluating prediction performance of individual lightweight CNN models, their simple one-stage ensemble, and the proposed two-stage NAS-driven ensemble strategy (NASMobV2). Baseline Models (One-Stage Training) Initially individual transfer learning (TL) models – NASNetMobile, MobileNetV2, MobileNetV3, ResNet50, VGG16, EfficientNetV2B1, and RegNetY002 were trained. The results (Table 7) showed that while ResNet50 underperformed (62.5% accuracy), lightweight CNNs such as MobileNetV2 (96.38%), NASNetMobile (95.0%), and RegNetY002 (94.5%) demonstrated strong predictive capability with relatively lower computation cost. MobileNetV3 performed comparably to NASNetMobile but failed to surpass MobileNetV2, hence it was not included in the ensemble.

Simple Ensemble (One-Stage Averaging):

To improve stability, the experimentation with simple averaging of MobileNetV2 and NASNetMobile predictions was performed. This ensemble achieved 97.8% accuracy. However, since averaging treats both models equally, the method did not leverage the superior predictive power of MobileNetV2.

Proposed Two-Stage NAS-Optimized Ensemble (NASMobV2):

To address the above limitation, a two-stage optimization strategy was implemented : Stage 1: Base models (NASNetMobile and MobileNetV2) were individually fine-tuned with modified classification layers. Stage 2: A neural architecture search (NAS), driven by Bayesian Optimization (Keras Tuner), was applied to optimize ensemble weights (ensuring weight1 + weight2 = 1) and the learning rate. This approach allows MobileNetV2 (higher-performing) to contribute more strongly, while still incorporating complementary features from NASNetMobile.

Comparative Results :

The final performance comparison is summarized below: Individual Models - Accuracy ranged between 95.0% – 96.38% (NASNetMobile, MobileNetV2). Simple Ensemble - Accuracy 97.8%. NAS-Optimized Ensemble (NASMobV2) -achieved 98.57% accuracy, 98.62% precision, and 98.25% recall, with an F1 score of 91, clearly outperforming both the simple averaging and individual models.

### Ablation study

To better understand the contribution of different components in the proposed NASMobV2 framework, an ablation study was conducted by systematically removing or isolating key design choices: (i) mutation of classification layers, (ii) ensemble learning, and (iii) NAS-based optimization with attention weighting.

**Table 14:** Ablation study results of different configurations.

Configuration	Mutation	Ensemble	NAS-based Weighting	Attention Mechanism	Accuracy (%)
MobileNetV2 (baseline)	✗	✗	✗	✗	95.12
MobileNetV3 (baseline)	✗	✗	✗	✗	95.37
NASNetMobile (baseline)	✗	✗	✗	✗	95.83
Modified Layers (mutation only)	✓	✗	✗	✗	97.10
Simple Averaging Ensemble	✓	✓	✗	✗	96.80
NAS-based Weighted Ensemble	✓	✓	✓	✗	98.00
Attention-based NASMobV2 (Proposed)	✓	✓	✓	✓	**98.57**

Baseline Models (MobileNetV2, NASNetMobile): Both individual transfer learning baselines achieved strong performance (>95% accuracy). However, they showed limitations in generalization under field-like validation conditions, with higher variance across nutrient deficiency categories.

Mutation of Classification Layers: By modifying the dense layers and introducing adaptive optimizers, an improvement was observed of  2–3% in validation accuracy compared to unmodified baselines. This step also reduced training instability.

Simple Averaging Ensemble: Combining MobileNetV2 and NASNetMobile through unweighted averaging further improved accuracy to  96.8%. This confirms that ensembling helps capture complementary features from the two backbones.

NAS-based Weight Optimization: Replacing manual or fixed averaging with Bayesian and Hyperband-based NAS optimization led to automatically learned dynamic weights (e.g., 0.45:0.55). This contributed an additional  1.5–2% gain, yielding 98.57% validation accuracy. This result highlights the effectiveness of the NAS weighting mechanism in adapting to feature distribution shifts.

**Fig. 16:**
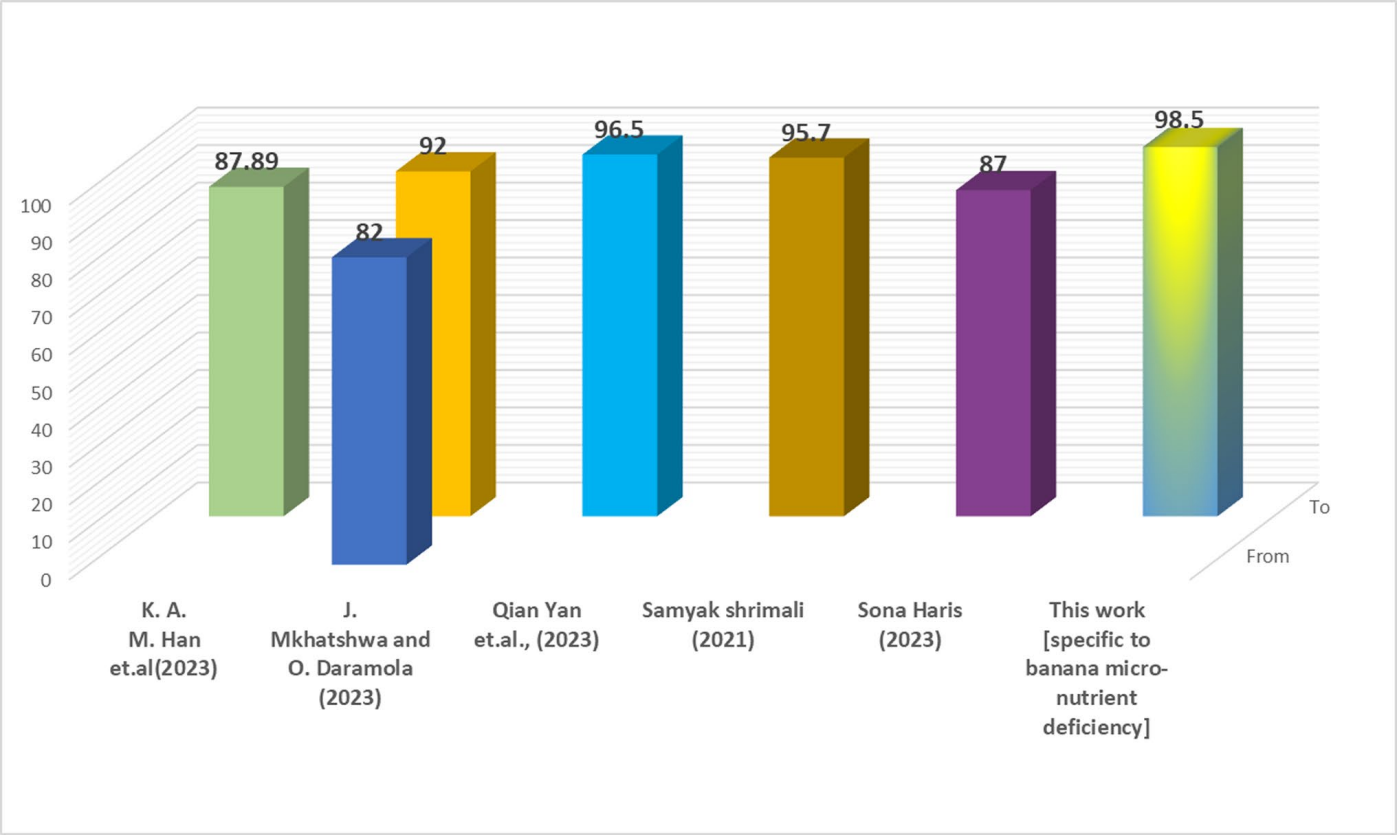
Similar contributions and their results.

**Fig. 17:**
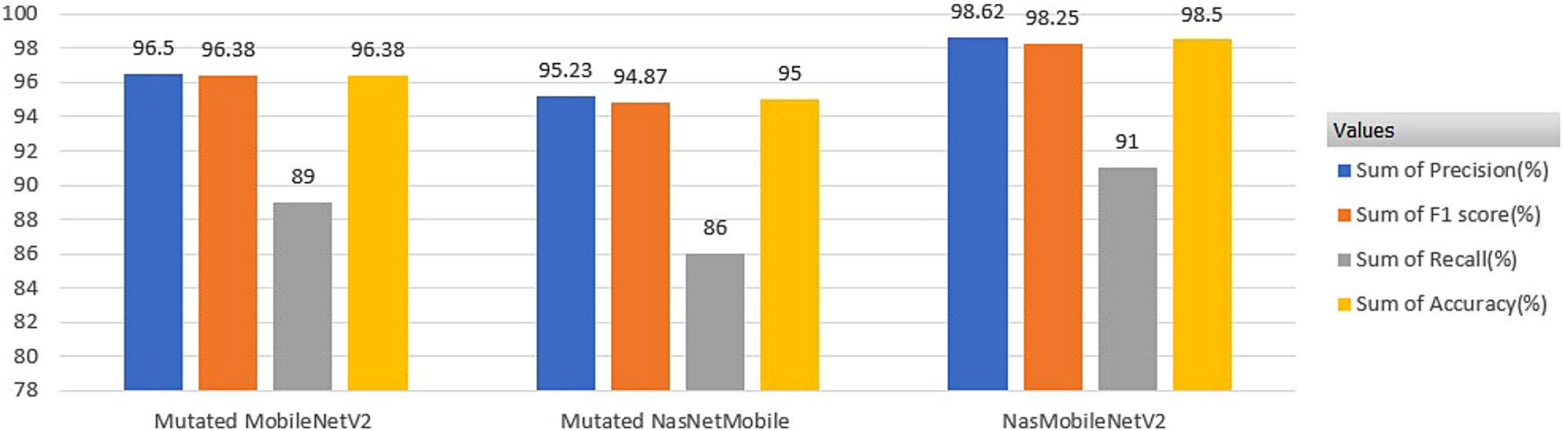
Comparison of Model Performances.

Attention-based NAS Ensemble: The final attention-driven NASMobV2 model achieved the best balance across metrics (precision, recall, and F1 score), with more stable predictions in minority classes (e.g., manganese deficiency). Table 14 summarizes the results showing the effect of mutation, ensemble, NAS-based weighting, and attention mechanism on accuracy. This demonstrates that the attention module plays a crucial role in dynamically emphasizing the stronger backbone depending on the input. The model visualization or explainability is shown as Fig. 15. Most studies use fixed weighting or simple averaging techniques in ensemble approaches for image classification with regard to crop nutrient deficiency. Here, in our research work of NAS driven mutated ensemble learning based model, contributes to optimal weights automatically rather than manual settings, adapting to different feature distributions and as a light-weight efficient performing model. Therefore, the proposed model achieves double-layer optimization by providing the best feature extraction and dynamically interpreting the ensemble weights, thus improving feature extraction in nutrient deficiency detection and classification. The Similar contributions from different authors are listed in Table 15, Fig. 16 and their comparison with our study is depicted in Fig. 17.

**Table 15:** Comparison of author contributions from similar works (2019–2025) and other STATE-OF-THE-ART (SOTA) methods on nutrient deficiency classification/leaf condition.

Authors (Year)	Crop & Classes	Methodology	Mode	Results (Acc / Prec / Rec / F1 / Sens / Spec)	Advantages / disadvantages
K.A.M. Han et al. (2023)^36^	Banana (8 classes + healthy)	ConvNeXTTiny	CNN	87.89 / NR / NR / NR / NR / NR	Adv: Lightweight CNN, good for multi-class banana dataset. Disadv: Limited accuracy, no deployment, crop-specific.
J. Mkhatshwa and O. Daramola (2023)^37^	Banana (3 classes)	Pretrained CNNs (VGG-16 / IncepV3)	CNN	82 / NR / NR / NR / NR / NR; 92 / NR / NR / NR / NR / NR	Adv: Transfer learning; improved accuracy. Disadv: Narrow scope (only 3 classes), not optimized for deployment.
Qian Yan et al. (2023)^38^	Banana dataset	MobileNetV3-CBAM	CNN	96.5 / NR / NR / NR / NR / NR	Adv: High accuracy with lightweight MobileNet + attention. Disadv: Lacks extended metrics, no app-level validation.
Sanyak Shrimali (2021)^8^	PlantVillage (14 crops)	Mobile App (PlantifyAI)	Mobile App	95.7 / NR / NR / 96.1 / NR / NR	Adv: Deployable app, broad crop coverage. Disadv: Not nutrient-specific, Relies on PlantVillage dataset (synthetic images, limited field realism).
Sona Haris (2023)^9^	Guava deficiency	CNN (mobile)	Mobile App	87 / NR / NR / NR / NR / NR	Adv: Focused nutrient detection. Disadv: Low accuracy, single crop validation.
Selvaraj et al. (2019)^20^	Banana, various parts	Tumaini app (CNN)	Mobile App	90–99 / NR / NR / NR / NR / NR	Adv: Real-world deployment. Disadv: Inconsistent results, Focused on disease/pests, not nutrient deficiencies; dataset limitations.
Bera et al. (2024)^39^	Banana & Coffee deficiencies	PND-Net (CNN+GCN)	DL	90.00 (Banana) / 90.54 (Coffee) / NR / NR / NR / NR	Adv: Region-aware GCN, cross-crop. Disadv: Moderate accuracy only.
Sunitha et al. (2024)^40^	Banana micronutrients	CNN + GUI	DL	$\sim$95 overall; Boron: 0.90/0.90/0.90; Iron: Prec=0.60 / NR / NR / NR	Adv: User-friendly GUI. Disadv: Poor performance on some nutrients.
Muthusamy et al. (2024)^41^	Banana micronutrient deficiency	IncepV3+Dense (ensemble)	DL	98.62 / 99.12 / 98.37 / 93 / NR / NR	Adv: High accuracy & F1; ensemble robust. Disadv: Missing fine-grain metrics.
Jeong et al. (2025)^42^	Soybean NPK deficiency	YOLOv8s (edge)	DL	NR / 0.929 / 0.877 / NR / NR / NR	Adv: Real-time edge deployment. Disadv: Limited metric reporting.
Araaf et al. (2024)*^43^	Coffee leaf rust	DL classifier	DL	95 / NR / NR / NR / NR / NR	Adv: Good disease detection. Disadv: Not nutrient-focused.
OIA (2024)*^44^	Coffee diseases	MobileNet	DL	97.3 / 0.563 / 0.571 / 0.567 / NR / NR	Adv: Benchmark comparison. Disadv: Low precision.
This Work (2025)	Banana deficiencies (B, Fe, Mn, Healthy)	NASMobV2 + Ensemble	Web, Mobile	**98.57** / **98.62** / **98.25** / **91** / /	Adv: **Significant accuracy with lightweight model, covers severity classification, multi-platform deployment**. Disadv: **Needs broader validation across multiple leaves/leaf occlusions**.

NR, not reported. Works marked * are disease-oriented but included as baseline leaf-condition references. NASMobV2 (this work) outperforms prior approaches in both accuracy and balanced metrics

### Limitations and future directions

This section outlines constraints such as the limited dataset size, potential bias due to controlled image capture conditions, and the need for testing under extreme field conditions (occlusion, overlapping leaves, varying lighting). We have also highlighted directions for future research to address these limitations. Although the proposed NASMobv2 model demonstrated strong performance (98.57%) in detecting boron, iron, and manganese deficiencies, a few limitations need to be acknowledged. First, the dataset–though extended during this study–was still restricted to a limited number of micronutrients and may not fully represent the wide variability in symptoms caused by environmental factors, crop varieties, and growth stages. Second, while the ensemble approach and NAS-based optimization improved classification accuracy, the model’s robustness on highly diverse field conditions, such as overlapping symptoms, mixed deficiencies, or low-quality images, remains to be validated. Third, the current implementation of the mobile and web applications successfully tested the model but still requires continuous updates for scalability, user-friendliness, and adaptability to new deficiencies. Looking ahead, future work will focus on expanding the dataset to include other important micronutrient deficiencies (e.g., copper, molybdenum) and validating the model across different crops and agro-climatic conditions. There is also a plan to integrate severity grading more deeply into the system to provide actionable insights for fertilizer management. As a future direction, there are plans to integrate SHAP-based explanations for complementing CAM methods. This will allow farmers not only to see where the model is focusing but also to understand the relative contribution of features, thereby increasing trust and transparency in the diagnosis. Finally, further enhancement of the mobile and web platforms–through real-time feedback, farmer-centered design, and decision-support tools–will ensure practical usability and wider adoption in precision agriculture.

### Ethical and social considerations

The integration of AI in agriculture has many benefits, but it also brings some ethical and social concerns. One important issue is the risk of wrong predictions, which could lead to poor nutrient decisions and cause crop loss or extra costs for farmers. To reduce this risk, focus on strong validation was conducted, clear comparisons with other models, and ablation studies to check the reliability of this approach. Another concern is data privacy. Since these systems often use farm images and location details, it is important to handle this information safely and protect farmers’ rights. Future use of such systems should follow responsible AI practices, including secure data storage, clear explanations of decisions, and tools that farmers can easily understand. By addressing these concerns, AI-based solutions in agriculture can become not only accurate but also trustworthy and fair, supporting sustainable farming practices.

## Conclusion

In this work, a lightweight attention-based deep learning model was proposed, optimizing via neural architecture search (NAS), for classifying micronutrient deficiencies in banana leaves (boron, iron, manganese) and distinguishing them from major diseases. By evaluating multiple mobile-friendly CNNs and incorporating dynamic attention in an ensemble, the NASMobV2 framework achieved 98.57% validation accuracy, consistently outperforming individual transfer learning models across precision, recall, and F1 score. The model further integrates severity estimation, enabling more actionable insights for early intervention. To ensure real-world utility, the system was deployed as both mobile and web applications, enabling rapid, in-field diagnostics with minimal computational demand. While validated on banana and extended datasets such as coffee leaves, future work should expand to additional deficiencies (e.g., copper, molybdenum), a wider set of diseases, and varied field conditions. Overall, the study demonstrates that NAS-optimized lightweight architectures with attention mechanisms can provide scalable, field-ready solutions for precision agriculture, supporting timely decision-making and sustainable crop yield improvement.

## Data Availability

An openly available repository (Mendeley dataset) was used to perform this study;(https://data.mendeley.com/datasets/7vpdrbdkd4/1), Request for any data or materials shall be addressed to the author(sudhakar.m2020@vitstudent.ac.in).
